# Alternative metabolic pathways and strategies to high-titre terpenoid production in *Escherichia coli*†

**DOI:** 10.1039/d1np00025j

**Published:** 2021-07-07

**Authors:** Mauro A. Rinaldi, Clara A. Ferraz, Nigel S. Scrutton

**Affiliations:** Manchester Institute of Biotechnology, Department of Chemistry, School of Natural Sciences, The University of Manchester 131 Princess Street Manchester M1 7DN UK nigel.scrutton@manchester.ac.uk

## Abstract

Covering: up to 2021

Terpenoids are a diverse group of chemicals used in a wide range of industries. Microbial terpenoid production has the potential to displace traditional manufacturing of these compounds with renewable processes, but further titre improvements are needed to reach cost competitiveness. This review discusses strategies to increase terpenoid titres in *Escherichia coli* with a focus on alternative metabolic pathways. Alternative pathways can lead to improved titres by providing higher orthogonality to native metabolism that redirects carbon flux, by avoiding toxic intermediates, by bypassing highly-regulated or bottleneck steps, or by being shorter and thus more efficient and easier to manipulate. The canonical 2-*C*-methyl-d-erythritol 4-phosphate (MEP) and mevalonate (MVA) pathways are engineered to increase titres, sometimes using homologs from different species to address bottlenecks. Further, alternative terpenoid pathways, including additional entry points into the MEP and MVA pathways, archaeal MVA pathways, and new artificial pathways provide new tools to increase titres. Prenyl diphosphate synthases elongate terpenoid chains, and alternative homologs create orthogonal pathways and increase product diversity. Alternative sources of terpenoid synthases and modifying enzymes can also be better suited for *E. coli* expression. Mining the growing number of bacterial genomes for new bacterial terpenoid synthases and modifying enzymes identifies enzymes that outperform eukaryotic ones and expand microbial terpenoid production diversity. Terpenoid removal from cells is also crucial in production, and so terpenoid recovery and approaches to handle end-product toxicity increase titres. Combined, these strategies are contributing to current efforts to increase microbial terpenoid production towards commercial feasibility.

## Abstract 2

Terpenoids are a diverse group of chemicals used in a wide range of industries. They can be made through alternative metabolic pathways to reach the higher needed for biomanufacturing.

## Graphical abstract

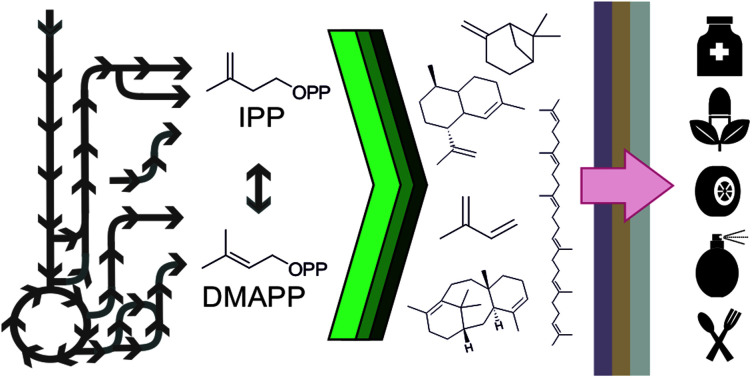

## 1 Introduction

Terpenoids form one of the largest and most diverse classes of chemicals comprising thousands of structures.^1^ They have a wide range of applications in multiple industries, ranging from pharmaceuticals such as anti-cancer drugs and antimalarials, to flavours and fragrances, and to agricultural products such as pesticides and insect repellents.^2^ These valuable chemicals are mostly made synthetically from petroleum or extracted from plants grown at agricultural scales. As the world economy turns away from fossil fuels and non-food uses for arable land, the need arises to develop microbial cell factories to produce terpenoids sustainably through fermentation of renewable feedstocks.

**Fig. 1:**
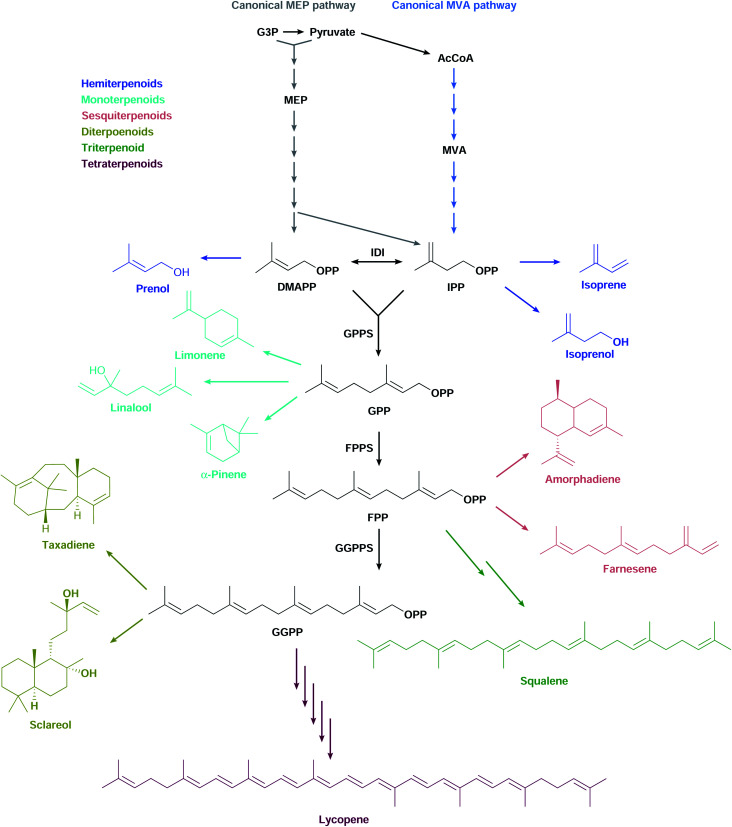
The canonical MEP and MVA pathways are the most commonly used to make diverse terpenoid structures.

Most terpenoids are made in nature from the interconvertible C5 precursors isopentenyl diphosphate (IPP)§§We use the correct name “diphosphate”, but the more frequently used abbreviations IPP, DMAPP, *etc.* and dimethylallyl diphosphate (DMAPP), produced from central metabolism *via* the 2-*C*-methyl-d-erythritol 4-phosphate (MEP) or the mevalonate (MVA) pathways (Fig. 1). IPP/DMAPP is used directly to make C5 hemiterpenoids or is combined with additional IPP/DMAPP molecules by prenyl diphosphate synthases (PPPS) to build longer prenyl diphosphates, such as C10 geranyl diphosphate (GPP), C15 farnesyl diphosphate (FPP), and C20 geranylgeranyl diphosphate (GGPP). Terpenoid synthases (TPSs) convert prenyl diphosphates into terpenoid backbones for C10 monoterpenoids, C15 sesquiterpenoids, C20 diterpenoids, C30 triterpenoids, or C40 tetraterpenoids, among others. These backbones are functionalised by additional enzymes that further expand the structural and chemical diversity of this class of compounds.

*Escherichia coli* is one of the main model organisms for terpenoid production, with vast physiological, biochemical and metabolic knowledge available as well as diverse engineering tools. *E. coli* is advantageous from an industrial perspective because it accepts a wide range of substrates and grows efficiently under low-cost and large-scale conditions.^3^ In general, engineered *E. coli* produces higher levels of hemi-, mono- and tetraterpenoids than other organisms, whereas engineered yeast strains produce higher levels of sesqui-, di- and triterpenoid compounds.^4–6^ Because *E. coli* is easy to manipulate, insights gained into the benefits of using this host and discussed in this review, can also be applied to more industrially relevant host bacterial organisms.

Advances in the last few decades have dramatically improved microbial terpenoid production. Only a few selected processes however are considered as having the potential to be marginally cost competitive. For example, microbial β-farnesene and semi-synthetic artemisinin production were demonstrated at industrial scales,^7,8^ but have not yet displaced traditional manufacturing routes that remain less expensive. Techno-economic analyses of microbial terpenoid production identify productivity or yield as a main cost driver and conclude titre improvements could enable commercialisation.^9,10^ Other cost intensive aspects are also a concern (*e.g.* capital infrastructure, feedstock provision, downstream processing amongst others).

Titres are the most commonly reported measure in metabolic engineering programs. Titres, however, can vary based on fermentation time and conditions, expression levels of genetic constructs, enzymatic activity of limiting steps, regulation points in the pathway, genetic background, carbon source and other factors.^11^ Varied production conditions have led to some of the highest reported titres for different terpenoids (Table S1†). Reported titres measured under different production conditions are not necessarily the best metric of the effectiveness of a pathway tested. However, a comparison of titres measured under different fermentation conditions available across the extensive terpenoid literature can still be useful to compare strategies for terpenoid production. In this review, we use titres to make direct comparisons (*i.e.* identical production conditions and within a single publication) whenever available. We review also titres obtained across different publications to gain insight into conditions that lead to the highest titres for a specific terpenoid. Our rationale is that different strategies that produce high titres are of interest from a learning perspective. Combinatorial approaches, for example, could potentially increase these titres further. In those cases where reported titres are low (*i.e.* lower than the highest reported titres for a specific terpenoid), the interpretation is more challenging but higher titres may become achievable pending further optimisation. In these cases, these lower titres provide an indication of how much optimisation remains to be done to approach the highest reported values in the literature.

Engineering the native MEP pathway or the heterologous MVA pathway in *E. coli* has enabled terpenoid production at the g L^−1^ scale (Table 1). Bottlenecks and regulation points in these canonical pathways have been overcome by overexpressing native genes or by using homologous enzymes from other organisms that are resistant to regulation or are more active. Using alternative pathways with higher orthogonality to native metabolism, and that avoid intrinsic regulation,^12^ potentially could lead to higher production titres. The use of alternative pathways can also avoid toxic intermediate build-up, bypass highly-regulated or bottleneck steps, and present more energetically efficient or shorter routes to the desired product. Such pathways are likely to be easier targets for optimisation through pathway engineering.

**Table 1:** Selected reports of the highest terpenoid titres produced from central metabolism in *E. coli*

Terpenoids and derivatives	Highest reported titre (g L^−1^ culture)^a^	Engineered precursor pathway	Reference
**Hemiterpenoids**			
Isoprene	60	MVA	76
Isoprenol	10.8	IPP bypass	127

**Monoterpenoids**			
Limonene	3.65^a^	MVA	160
-Perillyl alcohol	0.105	MVA	138
Sabinene	2.65	MVA	161
Geraniol	2.124	MVA	170
-Geranyl acetate	4.8	MVA	163
Linalool	1.523	MVA	307
α-Pinene	0.97	MVA	164
1,8-Cineole	0.653	MVA	165
-Hydroxycineole	0.056	MVA	308
Myrcene	0.058	MVA	166

**C11**			
2-Methylenebornane	0.014	MVA	146

**Sesquiterpenoids**			
Amorphadiene	30	MVA	172
-Artemisinic-11S,12-epoxide	0.25	MVA	173
-Artemisinic acid	0.105	MVA	174
Viridiflorol	25.7	MVA	172
β-Farnesene	10 0.67	MVA	175
(−)-α-Bisabolol	9.1	MVA	309
(+)-Isodauc-8-en-11-ol	1.16	MVA	184
α-Farnesene	1.1	MVA	176
α-Bisabolene	1.15	MVA	58
Farnesol	1.419	MVA	310
Protoilludene	1.119	MVA	311
Pentalenene	0.78	MVA	231
Epi-isozizaene	0.728	MVA	231
-Albaflavenol	0.013	MVA	308
-Albaflavenone	0.003	MVA	308
Cubebol	0.497	MEP	44
Guaia-6,10(14)-diene	0.468	MVA	312
Longifolene	0.382	MVA	182
Nerolidol	0.323	MVA	178
β-Copaene	0.215	MEP	44
(+)-Zizaene	0.211	MVA	179
Caryophyllene	0.1	MVA	229
-Caryolan-1-ol	0.01	MVA	229
α-Isocomene	0.0775	MVA	231
(−)-5-Epieremophilene	0.076	MVA	180
Valerenadiene	0.062	MVA	88
α-Humulene	0.06	MVA	313
(−)-Patchoulol	0.04	MVA	314
α-Copaene	0.007	MVA	181
Cadinene	0.0035	MVA	181
-8-Hydroxycadinene	0.06	MVA	174

**Diterpenoids**			
Sclareol	1.46	MVA	185
Taxadiene	1.02	MEP	40
-Taxadien-5α-ol	0.058	MEP	40
Levopimaradiene	0.7	MEP	186
*cis*-Abienol	0.22	MVA	183
Cembratriene-ol	0.079	MEP	45
Kaurene	0.032	MEP	187
Abietadiene	0.03	MVA	89

**Triterpenoids**			
Squalene	0.612	MVA	277
Dammarenediol-II	0.0086	MEP	315

**Tetraterpenoids**			
Lycopene	448 mg g^−1^ DCW^b^	MEP	41
-β-Carotene	3.6	MEP and MVA	316
- -Astaxanthin	1.18	MEP and MVA	316
- -Zeaxanthin	0.722	MVA	317
- -β-Ionone (C13)	0.5	MVA	318
- -Retinol (C15)	0.076	MEP and MVA	319
α-Ionone (C13)	0.48	MVA	318

**Other**			
Coenzyme Q10	0.00564	MEP	320

a Where titres were reported only for the organic phase, the organic phase titre was divided by the aqueous-to-organic-phase ratio.

b For reference, the next highest titre reported in g L^−1^ is 3.52 g L^−1^ or 50.6 mg g^−1^ DCW lycopene.^300^

In this review, we focus on both canonical and alternative pathways for terpenoid production in *E. coli* and discuss strategies to increase production titres to levels that might become commercially attractive. Also, we review ways to expand terpenoid diversity and to create new-to-nature industrially relevant molecules. Finally, we discuss methods used to increase the robustness of the production host and physical separation methods during fermentation to address problems associated with terpenoid end-product toxicity.

## 2 Canonical pathways to IPP and DMAPP

Most organisms use the classical MEP or MVA pathways to make terpenoids (Fig. 1). Bacteria use the MEP pathway, with the exception of Actinobacteria, Bacteroidetes, Chloroflexi, Firmicutes and Proteobacteria which have the classical MVA pathway or a variation thereof. Eukaryotes use the MVA pathway.^13,14^ In addition, plants use the MEP pathway in plastids, which is also essential for survival. Most archaea use alternative MVA pathways, except Sulfolobus, Metallosphaera and Acidianus which use the classical MVA pathway.^14^ Both the canonical MEP and MVA pathways have been engineered and opitimised to make terpenoids in *E. coli*.

### 2.1 The native MEP pathway

**Fig. 2:**
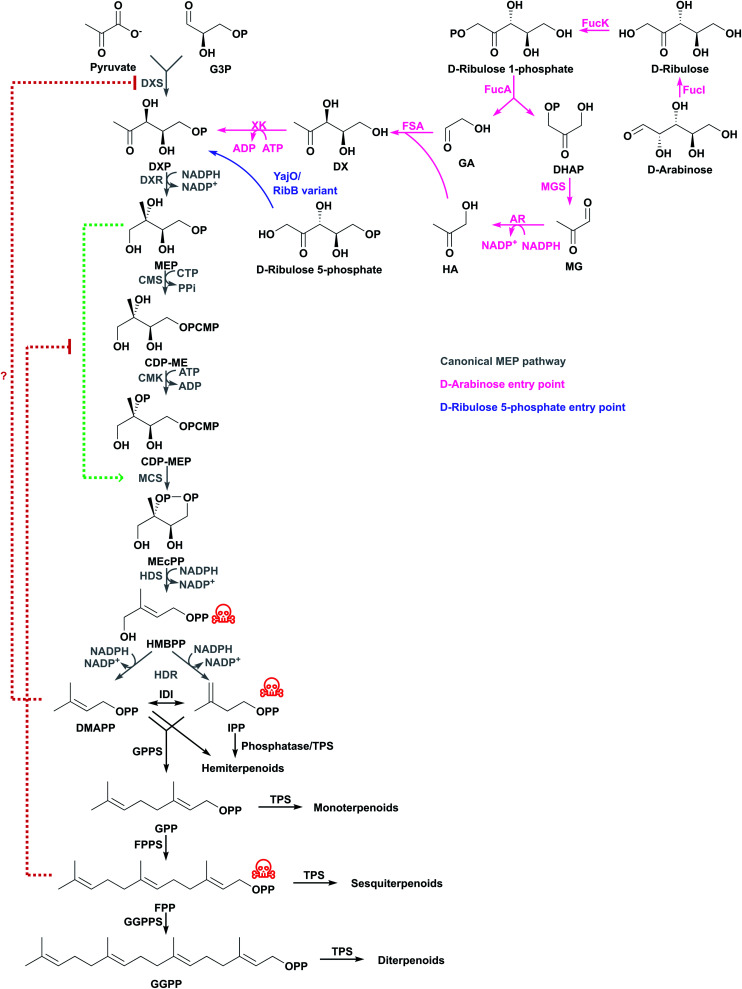
The canonical MEP pathway is the native pathway to terpenoids in *E. coli* and additional entry points have been tested for terpenoid production. Some intermediates are toxic (indicated with red skull and crossbones) and activate or inhibit enzymes in the pathway (indicated with green or red dashed lines, respectively). Question mark indicates regulation has not been confirmed in *E. coli*.

The MEP pathway starts with the condensation of pyruvate and d-glyceraldehyde 3-phosphate (G3P) from central metabolism to form 1-deoxy-d-xylulose 5-phosphate (DXP) catalysed by DXP synthase (DXS) in a thiamine diphosphate-dependent reaction (Fig. 2).^15,16^ DXP is converted to MEP by DXP reductoisomerase (DXR) using NADPH,^17^ and MEP is activated with cytidine 5′-triphosphate (CTP) to produce 4-diphosphocytidyl-2-*C*-methyl-d-erythritol (CDP-ME) by CPD-ME synthase (CMS).^18^ CDP-ME is phosphorylated by CDP-ME kinase (CMK), and cyclised to 2-*C*-methyl-d-erythritol 2,4-cyclodiphosphate (MEcPP) by MEcPP synthase (MCS).^19,20^ The cyclic diphosphate ring is opened and a reductive dehydration is catalysed by 4-hydroxy-3-methyl-butenyl diphosphate synthase (HDS) to produce 4-hydroxy-3-methyl-butenyl diphosphate (HMBPP).^21–23^ Finally, a reductive dehydration of HMBPP by HMBPP reductase (HDR) produces both IPP and DMAPP at a ratio of 5 : 1 in *E. coli*,^23–25^ and IPP and DMAPP are interconverted by IPP delta-isomerase (IDI). Because both IPP and DMAPP are produced by the MEP pathway, IDI is not essential in organisms containing the MEP pathway, and many bacteria do not have a gene encoding this enzyme.^26^

NADPH is consumed in the DXR, HDS and HDR steps.^27^ CTP is consumed at the CDP-ME step and ATP is consumed at the CMK step. Because CTP loses a diphosphate moiety, it needs 2 ATP molecules for regeneration. Therefore, the MEP pathway consumes 3 NADPH and 3 ATP for each IPP/DMAPP from pyruvate and G3P.^28^

#### 2.1.1 Regulation

The MEP pathway has several regulation points. Overexpression of native genes or expression of heterologous variants for these steps is one of the main strategies to increase terpenoid titres through this pathway.

DXS catalyses the most tightly regulated step. *Populus trichocarpa* DXS is feedback inhibited by IPP and DMAPP, which compete in the thiamine diphosphate binding pocket.^29^ This regulation is thought to occur in other species as well. Overexpressing *dxs* stimulates the MEP pathway and it leads to up to 10-fold titre increases;^30–33^ this has contributed to some of the highest reported terpenoid titres using the MEP pathway (Tables 1 and S1†).

Along with DXS, IDI is another important regulation point of the MEP pathway.^34^ As under normal growth conditions *idi* is expressed at low levels,^35^*idi* overexpression leads to higher terpenoid titres. For example, *idi* overexpression doubled isoprene production to 1 mg g^−1^ h^−1^ (ref. 36) and, together with *dxs* overexpression, increased limonene titres from 4.9 mg L^−1^ to 17.4 mg L^−1^.^37^

Optimisation of other steps in the MEP pathway might also be necessary to achieve higher terpenoid titres. Overexpressing *dxs*, *dxr*, and *idi* increases isoprene production 4.8-fold to 2.7 mg g^−1^ h^−1^.^36^ The insertion of a strong bacteriophage T5 chromosomal promoter controlling the genes encoding CMS and MCS, or CMK in *E. coli* increased β-carotene production 1.4- or 1.2-fold, respectively,^35^ suggesting these enzymes are also partially limiting. A potential reason why MCS is limiting might be that MEP activates MCS, and this feed-forward mechanism is inhibited by FPP (Fig. 2).^38^ Additional MCS might help to overcome this negative feedback mechanism.

Balancing expression of the genes that encode HDS and HDR increased β-carotene production by preventing accumulation of the toxic intermediate HMBPP and increasing MEcPP consumption before it effluxes from the cell.^34,39^ In one of the most successful attempts to engineer the MEP pathway in *E. coli*, a multivariate-modular pathway engineering approach was used to balance expression levels of DXS, CMS, CMK and IDI. This leads to the production of 1 g L^−1^ taxadiene in fed-batch fermentation.^40^ Similarly, overexpressing these genes and balancing the three remaining genes in the lycopene biosynthesis pathway leads to the highest reported lycopene titres.^41^

#### 2.1.2 Alternative genes

Using enzymes from other organisms at bottleneck steps in the pathway has been shown to be a useful strategy to increase terpenoid titres. Expressing *Bacillus subtilis dxs* doubles β-carotene titres in *E. coli* and use of the *B. subtilis* IDI homolog leads to a further doubling of β-carotene production.^42^ Also, expressing the *B. subtilis dxs* and *dxr* genes doubles isoprene titres compared to strains overexpressing the corresponding *E. coli* genes, achieving titres of 314 mg L^−1^.^31^*Bacillus licheniformis idi* also outperformed native *idi*, increasing lycopene titres from 288 to 352 mg L^−1^.^43^ Finally, *Haematococcus lacustris idi* was used in the highest titre reports for cubebol, β-copaene, and cembratriene-ol production (Table S1†).^44,45^

### 2.2 The MVA pathway

The *Saccharomyces cerevisiae* MVA pathway was the first alternative pathway to IPP/DMAPP to be introduced into *E. coli* and led to major improvements in reported titres.^46^ In part, these increases in reported titres are likely attributable to the MVA pathway improving IPP and DMAPP supply.^47^ This follows because the MVA pathway is mostly orthogonal to native *E. coli* metabolism.

**Fig. 3:**
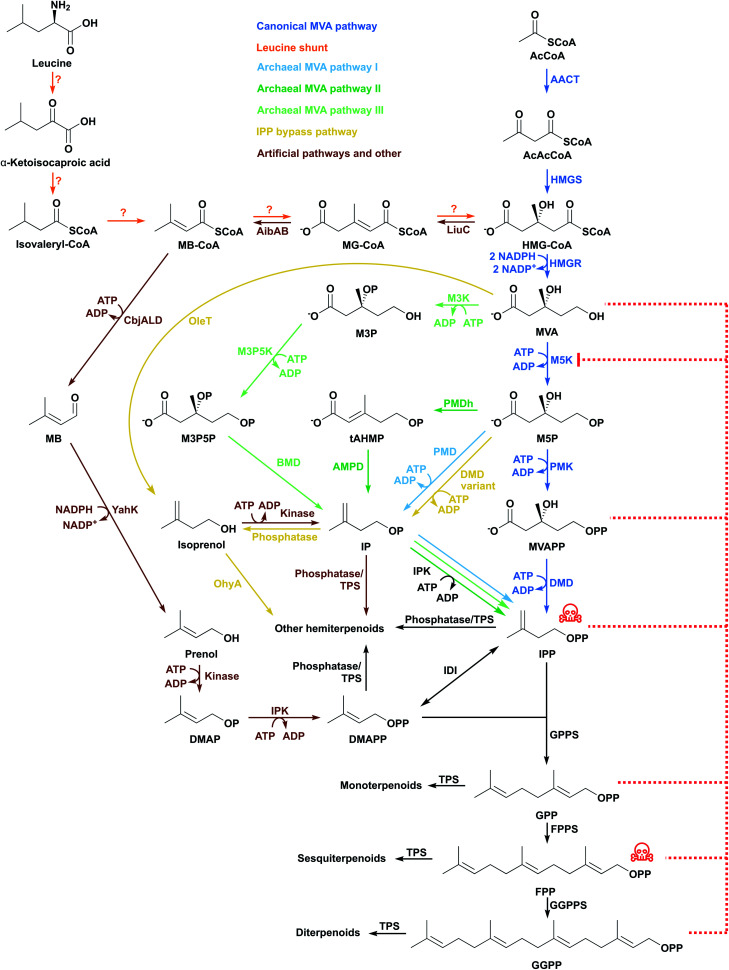
The MVA pathway and alternative pathways have been inserted in *E. coli* to produce terpenoids. Some intermediates are toxic (indicated with red skull and crossbones) and inhibit enzymes in the pathway (indicated with red dashed lines). Question mark indicates that an enzyme for the step has not been identified.

The MVA pathway starts with a Claisen condensation of two acetyl-CoA (AcCoA) molecules catalysed by an acetoacetyl-CoA thiolase (AACT), producing acetoacetyl-CoA (Fig. 3). The addition of a third AcCoA produces 3-hydroxy-3-methylglutaryl-CoA (HMG-CoA) in an aldol reaction catalysed by HMG-CoA synthase (HMGS).^48^ The reduction of HMG-CoA by HMG-CoA reductase (HMGR) with two equivalents of NADPH yields the 6-carbon intermediate MVA. MVA is phosphorylated twice in the 5-OH position by ATP, first by mevalonate 5-kinase (M5K) producing mevalonate 5-phosphate (M5P), and then by phosphomevalonate kinase (PMK), resulting in mevalonate diphosphate (MVAPP).^49–51^ This molecule is further decarboxylated in an ATP-mediated reaction catalysed by mevalonate diphosphate decarboxylase (DMD), producing only IPP.^50^ DMAPP is obtained from IPP by the action of IDI. The MVA pathway is conceptually divided into an upper pathway, from AcCoA to MVA, and a lower pathway, from MVA to IPP/DMAPP.

HMGR consumes 2 molecules of NADPH and M5K, PMK and DMD each consume ATP. In total, the MVA pathway therefore uses 2 NADPH and 3 ATP molecules to produce one molecule of IPP/DMAPP from AcCoA.

#### 2.2.1 Regulation

Heterologous MVA pathways usually produce higher terpenoid titres. That said, non-native MVA pathways can also affect a host's intrinsic regulatory mechanisms, causing an imbalanced consumption of precursors and resources, and therefore affecting cell fitness, growth and yield of a target terpenoid product.^52^ Therefore, fine-tuning relative expression of MVA pathway genes is necessary to reduce accumulation of known toxic intermediates such as HMG-CoA and IPP (Fig. 3), and to optimise potential bottleneck steps.^53,54^

The upper MVA pathway (from AcCoA to MVA) can be limiting in *E. coli* terpenoid production. MVA supplementation into the culture broth of cells harbouring the entire MVA pathway increases amorphadiene titres, suggesting that provision of mevalonate by the expressed pathway is a bottleneck.^52^ Fine-tuning HMGS expression is important as high levels of this enzyme can accumulate the cytotoxic product HMG-CoA,^52^ which inhibits fatty acid biosynthesis, membrane formation and cell growth.^55^ HMGS allocates carbon flux to the MVA pathway and low HMGS levels can cause metabolic carbon to be redirected to acetate production instead.^56^ Furthermore, HMGR catalyses the only enzymatic reaction of the mevalonate pathway that uses a redox cofactor. Overexpression of HMGR can therefore disrupt the cellular redox balance. Fine-tuning HMGR levels and/or supply of redox cofactors are potentially important strategies to optimise pathway performance.^57,58^

M5K is one of the most regulated enzymes of the MVA pathway and its activity can be inhibited by high levels of substrate and product (Fig. 3).^57,59–62^ In most organisms, downstream diphosphate intermediates such as IPP, DMAPP, GPP, FPP and GGPP inhibit M5K by binding competitively to the ATP-binding site of M5K.^60,63–65^*Streptococcus pneumoniae* M5K is inhibited by MVAPP,^66^ but *Staphylococcus aureus* M5K is not.^60^*S. aureus* and S. *cerevisiae* M5K are also inhibited by substrate at millimolar MVA concentrations.^57,60^ This might explain why MVA can be produced at 30 g L^−1^,^67^ whereas high level accumulation of metabolites downstream of this step in the MVA pathway is challenging. PMK was also identified as a bottleneck in an amorphadiene producing *E. coli* strain. Targeted proteomics indicated poor production of this enzyme in the cell. Codon-optimisation and use of stronger promoters to express the gene increased both enzyme and mRNA transcript levels, with concomitant improvements in amorphadiene titres.^68^

*E. coli* has basal levels of IDI activity. However, overexpression of *idi* leads to higher terpenoid production when using the MVA pathway. Basal IDI levels are relatively low and the MVA pathway requires IDI to balance IPP and DMAPP. Overexpression of IDI in a strain harbouring the MVA pathway was found to increase lycopene titres 10-fold to 22.2 mg L^−1^.^54^ Similarly, *idi* overexpression increases DMAPP supply and leads to increases in β-farnesene titres of almost 200-fold (8.74 g L^−1^).^69^

#### 2.2.2 Alternative genes

Recruiting better performing homologous enzymes from alternative organisms is often a good strategy to minimise bottlenecks in engineered MVA pathways. For example, recently identified feedback-resistant M5K homologs maintain high activity even in the presence of prenyl diphosphates and MVAPP.^65,70,71^ These homologs can reduce MVA accumulation and are valuable biocatalysts for terpenoid production compared to traditionally used M5K from *S. cerevisiae* and *S. aureus*.^72–75^ The highest isoprene titres achieved so far use the feedback-resistant *Methanosarcina mazei* M5K;^76^ (−)-α-bisabolol titres were increased 1.7-fold, reaching almost 600 mg L^−1^ by replacing *S. aureus* M5K with the *M. mazei* homolog.^73^ Protein engineering can also be used to improve substrate affinity, and thermal and pH stabilities of feedback-inhibited M5K. An *E. coli* strain harbouring the *S. cerevisiae* M5K_V13D/S148I/V301E_ variant produced 1.43 g L^−1^ lycopene, a titre that is 2.4-fold higher compared to the strain containing the wild-type homolog.^77^

Protein engineering has also been used to generate a catalytically superior *S. cerevisiae* IDI variant (L141H/Y195F/W256C), which improves lycopene production 1.8-fold, reaching 1.2 g L^−1^.^78^ Moreover, type II IDIs recently identified in some archaea,^79–81^ Gram-positive bacteria^82,83^ and cyanobacteria^84^ might also be beneficial in terpenoid production using the MVA pathway when compared to type I IDIs. These type II enzymes differ in amino acid sequence, structure, and reaction mechanism compared to the type I IDIs.^84^ Use of *B. licheniformis* type II *idi* in place of *E. coli idi* increased lycopene production (from 132 mg g^−1^ DCW to 181 mg g^−1^ DCW) in *E. coli* strains containing the MVA pathway.^43^ Use of *S. aureus* type II IDI also increased isoprene levels by 1.57-fold relative to *E. coli* strains containing the *S. cerevisiae idi*.^74^

Use of an *Enterococcus faecalis* HMGS variant (HMGS_A110G_) increases isoprene production 1.5-fold relative to strains containing the wild-type homolog, achieving production titres for isoprene of 6.3 g L^−1^.^85^ Furthermore, use of five different HMGR homologs has been investigated for amorphadiene production in *E. coli*.^57^*In vitro* assays showed that this enzyme can readily catalyse the reverse reaction. HMGR homologs with a high forward and low reverse reaction rate produced higher mevalonate levels *in vivo*. Nevertheless, when the MVA pathway harbouring these HMGR homologs was coupled to an amorphadiene producing cassette, the strains that accumulated more mevalonate did not produce the highest amorphadiene titres. The low titre obtained with the high MVA producing strains was attributed to substrate inhibition of M5K.^57^

In a more integrated approach, Liu *et al.* (2019) built an MVA pathway using *E. faecalis* AACT and HMGR, *M. mazei* M5K, and *S. pneumoniae* PMK, DMD and IDI for isoprene production.^53^ This pathway initially produced only 55.4 mg L^−1^ isoprene, but optimising promoter, plasmid copy number and the gene expression cassette, increased isoprene titres to almost 600 mg L^−1^.^53^ Replacing *S. cerevisiae* enzymes of the upper MVA pathway with *E. faecalis* homologs increased MVA accumulation 50-fold and isoprene production reached 500 mg L^−1^.^85^ Comparing the lower MVA pathways from *S. aureus*, *Streptococcus pyogenes*, *S. pneumoniae*, *E. faecalis*, and *S. cerevisiae* in a Coenzyme Q10 producing *E. coli* strain showed that *S. pneumoniae* genes led to a higher Coenzyme Q10 content under almost all conditions.^47^ In a similar study, the *S. pneumoniae*, *S. pyogenes*, *S. aureus*, *E. faecalis* and *S. cerevisiae* lower MVA pathways were compared to produce β-carotene supplying MVA as substrate. In this case, the *S. pneumoniae* and *S. cerevisiae* pathways were shown to be the best performers.^86^

The highest titres reported in *E. coli* for the production of most terpenoids – especially the hemi-, mono- and sesquiterpenoids – are obtained through heterologous expression and engineering of the MVA pathway. That said, the highest reported titres for some diterpenoids and lycopene have been obtained using optimised MEP pathways (Section 2.1 and Table 1). Higher titres are generally obtained using optimised MVA pathways rather than MEP pathways for isoprene,^87^ amorphadiene,^46^ valerenadiene,^88^ abietadiene,^89^ β-carotene,^42^ and retinoids^90^ production. These pathways can also be combined. Simultaneous engineering of both pathways leads to higher titres of the tetraterpenoid β-carotene than through the engineering of either pathway alone, leading to the highest reported β-carotene titres.^42^ Consistently, for lycopene production, inserting MVA pathways leads to further improvements in product titres compared to the use of MEP pathways alone.^91^ Furthermore, use of both the MEP and MVA pathways leads to a synergistic increase in isoprene titres to 24 g L^−1^, with flux increasing through both pathways, possibly because under oxygen-limiting conditions the MVA pathway produces reducing power from glucose that can be used by the MEP pathway.^92^ In contrast, inserting the MEP pathway into *S. cerevisiae* did not lead to major improvements in terpenoid titres, likely because *E. coli* HDS and HDR are expressed mainly as insoluble proteins in *S. cerevisiae*.^93^

## 3 Alternative precursor pathways

Canonical terpenoid pathways have been extensively engineered and optimised, but only a small number of terpenoids have been obtained at levels that might be considered as being attractive for commercial production. Variations of existing canonical pathways, or the development of fundamentally new precursor pathways, are alternative engineering approaches of potential benefit. Such strategies can give rise to higher degrees of orthogonality, or by replacing specific steps in existing pathways with metabolite shunts, bypass regulation points and/or avoid the accumulation of potentially toxic intermediates. Inclusion of alternative substrates and biocatalysts in biosynthetic pathways can also increase terpenoid diversity and lead to the production of compounds that are of potential commercial value (*e.g.* pharmaceutically active compounds, flavours, fragrances, and fuels) and that are readily accessed through modified terpenoid biosynthetic pathways.^94^

### 3.1 Alternative entry points to the MEP and MVA pathways

#### 3.1.1 Pathways to DXP

Because DXS is one of the most regulated steps in the MEP pathway, alternative pathways to DXP could potentially increase carbon flux towards terpenoid biosynthesis. The structural similarity between DXP and other pentose phosphates has inspired the use of two *E. coli* genes to convert d-ribulose 5-phosphate from central metabolism to DXP.^95^ Overexpressing *yajO* or a *ribB* variant rescues the ability of an *E. coli dxs* knockout strain to grow. The encoded proteins have been shown to produce DXP from d-ribulose 5-phosphate (Fig. 2).^95,96^ Overexpressing *yajO*, or expressing a *ribB* mutant, modestly increases bisabolene levels (from approximately 1 mg g^−1^ DCW to almost 3 mg g^−1^ DCW) in *E. coli*. Fusing the RibB variant to DXR further increases bisabolene content (to 9 mg g^−1^ DCW).^95^

More recently, another pathway to DXP from pentoses was developed to produce lycopene in *E. coli*. In this pathway, a first module of genes degrades exogenous d-arabinose to glycolaldehyde and hydroxyacetone, which are subsequently condensed by a second module consisting of fructose-6-phosphate aldolase to produce 1-deoxyxylulose. This latter compound is then promiscuously phosphorylated by xylulose kinase to DXP (Fig. 2). The engineered strains were reported to have a 4-fold increase in lycopene content with exogenous hydroxyacetatone, reaching almost 900 μg g^−1^ DCW lycopene.^97^

A natural MEP pathway shunt was discovered in *Rhodospirillum rubrum* as part of *S*-adenosylmethionine-dependent polyamine metabolism^98,99^ and later found in several other bacteria.^100^ In this route, DXP is produced in four enzymatic steps from 5′-methylthioadenosine, the dead-end product of universal polyamine metabolism. This shunt increased levels of native carotenoid-based pigments in *R. rubrum*^98^ possibly by increasing DXP levels, although DXP was not quantified. A similar pathway in pathogenic *E. coli* produces DXP from 5′-deoxyadenosine, a radical *S*-adenosylmethionine biproduct.^101^ These pathways could be exploited in non-pathogenic *E. coli* in attempts to increase terpenoid titres.

#### 3.1.2 Leucine shunt

In most organisms, leucine can be a substrate for terpenoid production by degradation to AcCoA and subsequent incorporation into the MVA pathway.^102,103^ However, in some organisms an alternative shunt pathway converts leucine to terpenoids.^103–105^ In the archaeon *Halobacterium salinarum* and the protozoan *Leishmania mexicana*, isotopic and stereoselectively-labelled leucine is assimilated into sterols and terpenoid-based lipids, respectively.^104,105^ These experiments validated a proposed route where leucine is converted in five steps to HMG-CoA, thus entering the MVA pathway (Fig. 3).^106^ To our knowledge, this shunt pathway has not been constructed in *E. coli* to support terpenoid production.

### 3.2 Archaeal MVA pathways

#### 3.2.1 Archaeal MVA pathway I

The lack of genes with sequence similarity to PMK, DMD and IDI in archaeal species suggests that these organisms do not have the canonical MVA pathway. In 2006 an alternative MVA pathway was elucidated by analysing the *Methanocaldococcus jannaschii* genome in detail.^107^ Isopentenyl phosphate (IP) was identified as the missing intermediate between M5P and IPP, and an isopentenyl phosphate kinase (IPK) was found to be responsible for the conversion of IP to IPP (Fig. 3). This suggests that the order of decarboxylation and phosphorylation is interchanged in this archaeon. An M5P decarboxylase (PMD) was later identified in the Chloroflexi bacterium *Roseiflexus castenholzii*, after *in vitro* assays with different substrates.^108^ The ATP-dependent PMD and IPK enzymes were then established as part of the first archaeal alternative MVA pathway.^109^ A patent application by Danisco and Goodyear describes the use of this alternative pathway in *E. coli*. Using the upper MVA pathway from *S. cerevisiae* and *Herpetosiphon aurantiacus* PMD and IPK leads to production levels for isoprene that are comparable to those using the classical *S. cerevisiae* MVA pathway in *E. coli*.^110^

#### 3.2.2 Archaeal MVA pathway II

A second variation of the MVA pathway in archaea was first identified in *Thermoplasma acidophilum* by two independent research groups^111,112^ and is likely present in at least eight closely related thermoplasmatales species for which genomic sequences are available.^113^ The pathway starts with the phosphorylation of MVA in the 3-OH position to produce mevalonate 3-phosphate (M3P) (Fig. 3). This step is performed by mevalonate 3-kinase (M3K), an enzyme homologous to decarboxylases of the MVA pathway. M3P is further phosphorylated to mevalonate 3,5-bisphophate by mevalonate 3-phosphate 5-kinase. The phosphate group (esterified at the 3-OH position) is removed during the decarboxylation performed by mevalonate biphosphate decarboxylase (BMD), producing IP. Although both decarboxylases in archaeal MVA pathways I and II share the same Asp/Lys/Arg catalytic triad, BMDs do not have ATP binding residues, and do not require ATP for activity.^113^ In the last step, IP is phosphorylated by an IPK to produce IPP,^111,112,114^ as also seen in the archaeal MVA pathway I.

The use of two different specialised enzymes (M3K and BMD) to catalyse a decarboxylation step performed by a single enzyme (DMD in the canonical MVA pathway and PMD in the archaeal MVA pathway I) could have arisen from an adaptation to extremely acid environments that did not favour the canonical pathway.^113^ DMD and PMD phosphorylate the hydroxyl in the 3-C position of MVAPP and M5P to produce IPP and IP, respectively.^113,115,116^ PMD loses kinase activity at low pH, but the decarboxylase activity remains intact, becoming a BMD.^113^ Therefore, there was potentially a need to evolve two separate enzymes that are homologous to PMD (M3K and BMD) to give rise to this alternative pathway.^113^ Of interest is that the phosphorylation in the 3-OH position and the decarboxylation of M3P are not sequential steps catalysed by the same enzyme (as is the case in the archaeal MVA pathway I) but rather are catalysed by two enzymes. There is also an additional step, a phosphorylation at the 5-OH position that needs a third enzyme from a different family.^117^

This pathway requires one more catalytic step compared to the canonical pathway, but has the same ATP requirement. As discussed above, the phosphorylation of MVA by M5K in the classical MVA pathway is one of the most heavily regulated enzymatic steps in the pathway.^63^ Consequently, an alternative pathway that by-passes this enzyme is of potential interest for terpenoid production. M3P accumulates in *T. acidophilum* and could be used to increase terpenoid production by heterologous expression of this alternative MVA pathway in *E. coli*.

*Picrophilus torridus* M3K was also used among other homologs to decarboxylate MVA and produce isoprenol *in vivo* and *in vitro* without needing to pass through IPP as an intermediate.^118^ In a different strategy, M3K was converted into a mevalonate 3-phosphate 5-kinase *via* a single amino acid substitution, and used with the archaeal MVA pathway I to produce approximately 0.347 μg lycopene per mg wet cells. This value is comparable to 0.491 μg lycopene/mg wet cells using the *S. cerevisiae* classical MVA pathway.^119^

A complete archaeal MVA pathway II has not been tested in *E. coli*. This would be of interest as it lacks one of the most regulated steps found in the canonical MVA pathway. Moreover, it would also be of interest to express both the archaeal MVA pathway II and the canonical MVA pathway together to explore any synergistic effects. M3K consumes MVA, and could thus potentially relieve substrate inhibition of M5K.

#### 3.2.3 Archaeal MVA pathway III

The lack of PMD and DMD homologs in most archaea has encouraged continued searches for alternative MVA pathways in these organisms. Recently, two new enzymes were discovered in the archaeon *Aeropyrum pernix*, mevalonate 5-phosphate dehydratase and *trans*-anhydromevalonate 5-phosphate decarboxylase, responsible for converting M5P to IP through a new intermediate, *trans*-anhydromevalonate 5-phosphate (Fig. 3).^120^ In the last step, IP is converted to IPP by IPK as in the archaeal MVA pathways I and II.

This pathway comprises enzymes which are not homologous to either mevalonate monophosphate or diphosphate decarboxylases, and seems to be widely conserved among archaea, including the taxonomically distant archaeon *M. mazei*.^14,121^ These enzymes do not require ATP. This might therefore create a more energy efficient route to terpenoids. *M. mazei* and *A. pernix* genes for the lower part of the archaeal MVA pathway III were introduced in *E. coli* to produce lycopene from exogenous mevalonolactone.^121^ Cells were cultivated semi-anaerobically because mevalonate 5-phosphate dehydratase is sensitive to oxidation, and produced up to 6 mg/OD_600_ lycopene.

There are very few examples of heterologous expression of any of the archaeal MVA pathways in *E. coli*. A lack of genetic tools for archaea may have limited studies of these pathways to *in vitro* assays.^14^ Nevertheless, these pathways may offer important advantages in terpenoid production compared to the classical MVA pathway. IPK is also present in plants and regulates carbon flux between the MEP and MVA pathways, balancing IP/IPP and DMAP/DMAPP ratios and helping to increase terpenoid production.^13,122^ Because IPP is toxic and inhibitory^123^ – as might also be the case with these other related compounds – it is possible that IPK could help reduce the effects caused by their accumulation. Other benefits of using archaeal variations compared to the canonical MVA pathway include bypassing highly regulated enzymes (archaeal MVA pathway II) and reducing ATP consumption (archaeal MVA pathway III).

### 3.3 IPP-bypass pathways

IPP accumulation is undesirable^46^ because it is converted to a toxic prenyl-ATP analog,^123^ and therefore strategies that bypass IPP can be beneficial by preventing IPP accumulation. Additionally, IPP-bypass pathways can reduce the number of metabolic steps and avoid competition for IPP with native metabolism and negative-feedback regulation. For example, combining the upper MVA pathway with a bacterial *Jeotgalicoccus* sp. ATCC 8456 fatty acid decarboxylase and a bacterial *Elizabethkingia meningoseptica* oleate hydratase led to isoprene production in *E. coli*.^124^ In this shorter pathway, MVA is decarboxylated directly to isoprenol (Fig. 3), which is further dehydrated to isoprene, thus allowing production of up to 620 mg L^−1^ isoprene using only two enzymatic steps from MVA (instead of five). This approach led to improved terpenoid levels, exceeding the highest titre achieved using the MEP pathway (314 mg L^−1^),^31^ but substantially lower than the highest titre achieved using a canonical MVA pathway (60 g L^−1^).^76^ Nonetheless, a shorter pathway could be beneficial when subjected to further titre optimisation (*e.g.* flux balancing and engineering).

Similarly, new pathways that utilise decarboxylation of M5P by a variant DMD (to produce IP) and endogenous phosphatases (to dephosphorylate IP into isoprenol) have been reported (Fig. 3).^125–127^ Isoprenol production at up to 10.8 g L^−1^ has been achieved in fed-batch cultures, the highest titre reported to date for this compound.^126,127^

Although promising for hemiterpenoids, these reduced MVA pathways have not been exploited for longer terpenoid products because they bypass the common building blocks IPP and DMAPP. Because TPSs require IPP/DMAPP and their derivatives, these are the only orthogonal pathways to terpenoids that branch before IPP/DMAPP.^128^

### 3.4 Isoprenol/prenol to IPP/DMAPP pathways

A novel and simpler three-step pathway which uses isoprenol or prenol as feedstocks to produce IPP and DMAPP was recently exploited by several groups.^129–134^ This pathway decouples terpenoid biosynthesis from central metabolism and therefore has the potential to increase metabolic flux towards the production of terpenoids.^135^ The pathway also reduces any negative impacts on cell growth, since glucose (or other carbon source used for bacterial growth) is reserved for primary metabolism.^136^ The first of the three steps in this pathway involves phosphorylation of exogenously added isoprenol or prenol to produce IP or DMAP. These are then converted by IPK to IPP or DMAPP, respectively (Fig. 3). Finally, IDI can be used to balance IPP and DMAPP levels.

Because the first reaction has not been found in existing metabolic pathways, several enzymes were tested to identify promiscuous kinase activity towards isoprenol and prenol. This new pathway was developed using *S. cerevisiae* choline kinase to catalyse the first phosphorylation reaction, *Arabidopsis thaliana* IPK and *E. coli* IDI.^129^ The pathway has been coupled to different downstream operons to produce several mono-, sesqui- and diterpenoids and lycopene, achieving a maximum titre of 9 mg g^−1^ DCW lycopene and 4.5 mg L^−1^ taxadiene,^129^ and 33.4 mg L^−1^ linalool.^137^ In a different study, *Shigella flexneri* non-specific acid phosphatase was used to catalyse the initial phosphorylation, and combined with *T. acidophilum* IPK and *E. coli* IDI to produce 190 mg L^−1^ lycopene.^133^ The highest reported terpenoid titres with this shorter 3-step pathway were obtained using *E. coli* hydroxyethylthiazole kinase, archaeal *Methanothermobacter thermautotrophicus* IPK and *Streptomyces* sp. IDI producing 2 g L^−1^ of geranoids and 0.5 g L^−1^ of limonene.^130^ The same authors also developed an alternative pathway comprising 8 enzymatic steps and requiring 2 ATP and 2 NAD(P)H (a lower cofactor requirement than for the canonical MVA or the MEP pathways) to obtain DMAPP from AcCoA, with isoprenol and prenol as intermediates. This new pathway produces 0.6 g L^−1^ of geranoids.

Titres obtained with isoprenol and prenol-derived pathways are generally lower compared to canonical pathways. However, in selected cases titres are promising and higher than those reported using canonical pathways.^37,138^ Shorter and orthogonal pathways are less regulated. This makes them easier to optimise compared to the canonical MEP and MVA pathways, which have been iteratively optimised over the past two decades. Consequently, the use of fed-batch processes and relatively simple pathway improvements may lead to competitive production strains after only a few rounds of pathway/strain optimisation.

### 3.5 Terpenoids outside the ‘isoprene rule’

**Fig. 4:**
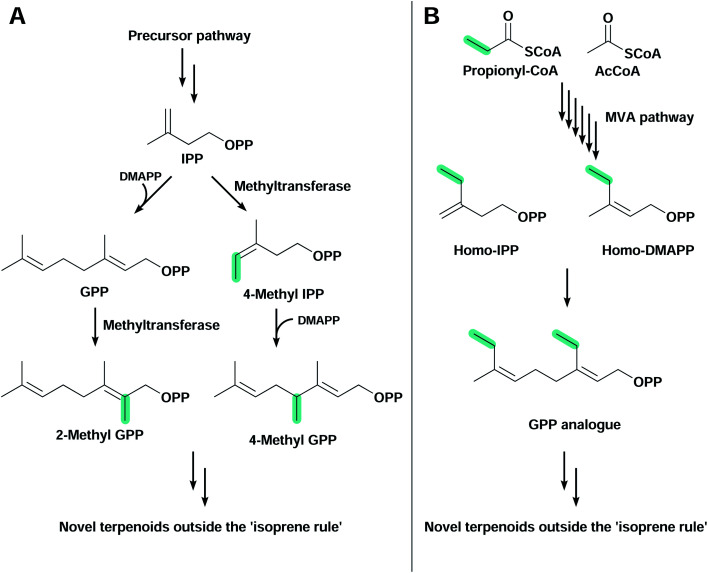
Strategies to produce terpenoids outside the ‘isoprene rule’. (A) Prenyl diphosphates such as IPP and GPP produced through any precursor pathway can be methylated by methyltransferases to produce 4-methyl-IPP or 2-methyl GPP, respectively. (B) The homomevalonate pathway present in some insect species starts with the condensation of propionyl-CoA and acetyl-CoA. The pathway produces the C5 diphosphate analogues homo-IPP and homo-DMAPP, which can be elongated to form higher prenyl diphosphates. Both strategies can lead to novel terpenoids with a flexible number of carbon atoms in their structure.

The large majority of terpenoids comprise C5 modules and follow the so-called ‘isoprene rule’,^139^ but recently discovered modifications in canonical pathways have allowed the biosynthesis of terpenoids outside this rule (Fig. 4). These orthogonal pathways allow the introduction of a flexible number of carbon atoms in terpenoid structures, further expanding chemical diversity. Simple modifications (*e.g.* methyl group addition) can improve biological activity,^140^ or generate novel organic structures that are otherwise difficult to obtain.

The C11 terpenoids 2-methylisoborneol and 2-methylenebornane, are responsible for the muddy smell in contaminated drinking water, and are derived from the 2-methylgeranyl diphosphate intermediate, a product of the electrophilic methylation of GPP^141^ catalysed by a *S*-adenosyl-l-methionine-dependent methyl transferase (Fig. 4A).^142,143^ Other methyl transferases capable of methylating IPP^144^ and FPP^145^ have also been found in nature and play an important role in the synthesis of non-conventional terpenoids. In *E. coli*, the expression of wild-type and variant 2-methylisoborneol and 2-methylenebornane synthases with a GPP methyl transferase and the MVA pathway have allowed the production of several known and unknown flavours and fragrances compromising mono-, C11-, sesqui- and C16 terpenoids.^146,147^ Another study used an IPP methyl transferase from *Streptomyces monomycini* to produce a variety of C6 and C7 IPP analogues in *E. coli* containing the MVA pathway. This has led to the production of several C11, C12, C16 and C17 terpenoids, and new-to-nature methylated carotenoids when co-expressed with β-carotene or zeaxanthin biosynthetic pathways.^148^

The second alternative pathway found in nature that is capable of producing terpenoids outside the isoprene rule is the homomevalonate pathway, used by insects to produce C16, C17 or C18 terpenoid hormones (Fig. 4B).^149–151^ Instead of starting the MVA pathway with the condensation of two AcCoA molecules, in this orthogonal pathway propionyl-CoA is condensed with AcCoA, producing an unusual ethyl branch in terpenoid skeletons instead of the conventional methyl branch.^152^ This homomevalonate pathway was recently expressed in *E. coli* to produce C16 terpenoids.^153^ Although C16 terpenoids were produced at low titre, this approach has the advantage of not requiring additional ATP or the regeneration of *S*-adenosyl-l-methionine, which is a feature when using methyl transferases.^153^

The recently developed pathways from isoprenol and prenol have also been used to produce non-conventional terpenoids by using similar alcohols as substrates. An *in vitro* study explored the substrate promiscuity of *E. coli* hydroxyethylthiazole kinase and *M. jannaschii* IPK on 15 isoprenol and prenol analogues.^134^ These enzymes are able to produce diphosphates from alcohols with up to seven carbons, and the addition of a PPPS and TPSs produce a number of known and new terpenoids.^134^

## 4 Alternative prenyl diphosphate synthases

PPPSs elongate terpenoid chains by catalysing sequential condensations of allylic diphosphates (DMAPP, GPP, FPP and GGPP) with IPP. In *E. coli*, the same PPPS, IspA, catalyses GPP and FPP synthesis consecutively and thus favours production of FPP over GPP.^154,155^ FPP might be favoured because several native *E. coli* terpenoids are sesquiterpenoids involved in vital cellular processes. For example, FPP is used in the biosynthesis of heme O of cytochrome *bo*, part of the respiratory chain.^156^ Also, FPP is elongated with IPP to make the essential C40 octaprenyl diphosphate, used in the biosynthesis of the side chains of ubiquinone and menaquinone, which are components of electron transfer systems in the respiratory chain.^157^ Furthermore, FPP is elongated with IPP to make the C55 undecaprenyl diphosphate, used in the biosynthesis of peptidoglycans, which are essential cell wall constituents.^158^

Heterologous PPPSs are used to increase monoterpenoid production in *E. coli*. Plant *Abies grandis* GPP synthase (AgGPPS) is the PPPS that leads to the highest monoterpenoid titres in *E. coli* (Table S1†).^138,159–166^ Unlike IspA, AgGPPS makes only GPP,^167^ and therefore likely increases the GPP pool available to monoterpenoid synthases. Thus, expressing *AgGPPS* leads to higher sabinene, pinene, limonene, or linalool titres compared to titres obtained by overexpression of *ispA*.^37,159,161,164,168^ Consistently, using a variant of IspA to favour GPP accumulation leads to higher titres for 1,8-cineole and linalool.^165^ Also, expressing this mutant *ispA* leads to higher pinene or linalool levels than overexpressing wild-type *ispA*, and to similar pinene levels to expressing *AgGPPS* in direct comparisons.^159,168^ AgGPPS also outperforms other GPPSs in direct comparisons^159,169,170^ and performs similarly to bacterial *Streptomyces* sp. strain KO-3988 GPPS.^171^ In one of the highest limonene titres reported, soluble AgGPPS levels did not correlate with titres, suggesting GPPS was not limiting.^171^

IspA is the most common PPPS used in those studies reporting the highest obtained sesquiterpenoid titres^58,172–181^ (Table S1†). IspA outperformed *Blakeslea trispora* and *S. cerevisiae* FPP synthase (FPPS) in direct comparisons.^182^ However, in two instances, *S. cerevisiae* FPPS outperformed IspA.^183,184^ ScFPPS is less catalytically efficient than IspA and this might be a reason for why IspA is often selected over ScFPPS.^183^ Several plant GGPP synthases (GGPPS) have also been used in some of the highest reported diterpenoids titres^40,45,89,185–187^ (Table S1†).

### 4.1 *cis*-Prenyl diphosphate synthases

**Fig. 5:**
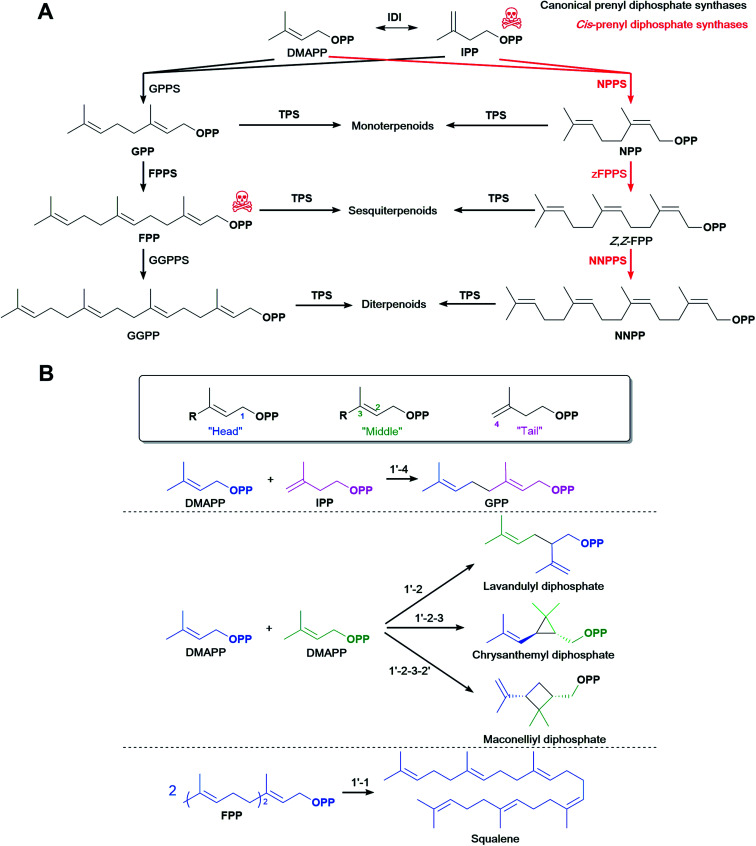
Alternative prenyl diphosphate synthases. (A) The *cis*-prenyl diphosphate pathway can be used to divert carbon flux to the terpenoid of interest. (B) Alernative prenyl diphosphate synthases catalyse non-head-to-tail condensation of isoprene units.

Most commonly, terpenoid biosynthesis uses *trans*-prenyl diphosphates produced by *trans*-PPPSs. Alternative *cis*-prenyl diphosphates are products of *cis*-PPPSs which were discovered in plants. These were recently used to create orthogonal pathways for microbial terpenoid production. In tomato, IPP/DMAPP is also converted to neryl diphosphate (NPP), the *cis*-isomer of GPP, then to *Z*,*Z*-FPP, the all-*cis*-isomer of the more common all-*trans E*,*E*-FPP, and then to nerylneryl diphosphate (NNPP), the all-*cis*-isomer of GGPP (Fig. 5A). Likewise, *cis*-specific TPSs convert these precursors to several terpenoids.^188–195^

Other *cis*-PPPSs and *cis*-specific TPSs were later characterised in other plant species,^196,197^ expanding the genes available for a combinational approach. That said, most canonical TPSs tested also accept *cis*-prenyl diphosphates,^75,198–203^ suggesting that canonical TPSs and their wide range of products are still available for use with *cis*-PPPSs. Variants of plant *Citrus limon* limonene synthase have higher affinity for NPP than the wild type,^198^ which suggests that canonical TPSs could be engineered to increase NPP affinity and further increase titres. *cis*-PPPSs can help divert metabolic flux towards the end product of interest, especially when the TPS might be the limiting factor owing to low catalytic activity and ineffective competition for *trans*-prenyl diphosphates.

This alternative pathway using *cis*-PPPSs was explored in yeast to increase monoterpenoid production. In *S. cerevisiae*, terpenoid biosynthesis might also favour production of essential FPP-derived terpenoids such as squalene, a yeast membrane component and major terpenoid carbon sink.^198^ Thus, monoterpenoid synthases compete with FPP production for GPP, and indeed monoterpenoid production is lower in *S. cerevisiae* than in some other organisms.^4^ Because the native *S. cerevisiae* FPPS cannot use NPP as a substrate,^198^ expressing *Solanum lycopersicum* NPP synthase (SlNPPS) diverts the metabolic flux from IPP/DMAPP to monoterpenoids instead of to FPP (Fig. 5A). Although SlNPPS does not accept IPP/DMAPP as efficiently as *S. cerevisiae* GPPS/FPPS and canonical TPSs have lower affinity for NPP than for GPP, monoterpenoid production is higher with SlNPPS. This suggests that making NPP increases monoterpenoid titres by creating an orthogonal pathway that effectively diverts metabolic flux. Indeed, assays using ^13^C-labelled substrates confirmed that most of the limonene, the intended monoterpenoid product in this case, is derived from NPP, whereas most of the FPP is derived from GPP.^198^ Consistent with this, a similar study produced higher limonene titres and lower squalene levels with NPPS than with a GPPS/FPPS variant that favours GPP in a direct comparison.^199^ Expressing NPPS also leads to high limonene titres in the yeast *Yarrowia lipolytica*.^204^

Competition with FPP in *E. coli* might not be as strong as in *S. cerevisiae* because generally higher monoterpenoid titres are achieved in *E. coli*.^4^ However, expressing SlNPPS in *E. coli* led to 694 mg L^−1^ limonene, 2.9-fold more than when expressing AgGPPS (181 mg L^−1^).^75^ This suggests that either the NPP pathway enzymes are more active, which is not the case in yeast,^198^ or alternatively that NPP bypasses competition with FPP for GPP in *E. coli*. Further optimisation showed that SlNPPS is efficient enough to sustain limonene production in *E. coli* to as much as 1.29 g L^−1^ limonene,^75^ an amount comparable to the highest reports obtained with other strategies.^58,138,160,171^ Thus, NPPS can sustain at least as much production as other approaches, and could produce higher titres under different conditions.

A *Mycobacterium tuberculosis Z*,*E*-FPPS was used in *E. coli* to produce *Z*,*E*-farnesol from exogenous MVA. *Z*,*E*-FPP is likely not incorporated into native *E. coli* metabolism, therefore creating an orthogonal pathway.^205^ Also, *S. lycopersicum* NNPP synthase (NNPPS) and lycosantalonol synthase were used in *E. coli* to demonstrate biosynthesis of the diterpenoid lycosantalene.^188^ Beyond these examples, *cis*-prenyl diphosphates longer than NPP have not yet been explored in *E. coli* or compared to *trans*-prenyl diphosphate pathways. However, they could be useful for the production of longer terpenoids if the native PPPSs and TPSs are *trans*-specific; the *cis*-prenyl diphosphates can then redirect carbon flux to the terpenoid of interest (Fig. 5A).

### 4.2 Non-head-to-tail prenyl diphosphate synthases, terpenoid cyclases and other prenyltransferases

The most common way of elongating prenyl diphosphate chains is in a 1′-4 head-to-tail fashion, referred to as ‘regular elongation’, where the allylic diphosphate precursor (such as DMAPP) is first ionised to form a carbocation and then undergoes a nucleophilic attack by the double bond of IPP followed by proton elimination to form the elongated allylic diphosphate.^206^

Some PPPSs catalyse alternative reactions to form non-head-to-tail connected terpenoid skeletons (Fig. 5B). These reactions, termed ‘irregular’, include head-to-head (1′-1) elongation, head-to-middle (1′-2) branching, cyclopropanation (c1′-2-3) or cyclobutanation (c1′-2-3-2′) reactions and are most commonly involved in the synthesis of some irregular longer terpenoids such as the triterpenoid squalene and C40 phytoene, the precursor of carotenoids.^207,208^ Shorter terpenoids formed in a non-head-to-tail fashion are very rare, but mono- and sesquiterpenoids with these irregular structures are found for example in plants^209^ and marine bacteria.^210^ A plant *Lavandula x intermedia* PPPS catalyses a head-to-middle condensation of two DMAPP molecules to generate lavandulyl diphosphate, the precursor of (*R*)-lavandulol and (*R*)-lavandulyl acetate.^209,211^ Also, a plant *Chrysanthemum cinerariaefolium* PPPS catalyses the cyclopropanation of two DMAPP molecules to form the irregular diphosphate chrysanthemyl diphosphate.^209,212^ Other irregular short diphosphates include cyclolavandulyl, maconellyl, planococcyl and isosesquilavandulyl diphosphates and are converted to short irregular terpenoids.^213^ To the best of our knowledge, *E. coli* has not been used as a platform to produce short terpenoids from non-head-to-tail condensations. Expressing and engineering these alternative PPPS could potentially create orthogonal terpenoid biosynthetic pathways similar to the examples listed above with *cis*-PPPS, and further diversify terpenoid molecules.

Terpenoid cyclases promote changes in terpenoid structure by catalysing reactions which generate multiple rings and stereocentres.^214^ Recently, several class I terpenoid cyclases were also found to be bifunctional and able to work as aromatic prenyltransferases. These enzymes catalyse the condensation of DMAPP or GPP and indole to produce prenylated indole compounds in *E. coli*. This activity might be more widely spread among terpenoid cyclases, opening up a strategy to decrease the accumulation of toxic phosphorylated intermediates such as DMAPP.^214^ A distant relationship between prenyltransferases and terpenoid cyclases was identified recently that revealed a new family of prenyltransferases, which uses a repurposed terpenoid cyclase structural fold to prenylate glutamic acid.^215^

Other prenyltransferases generated different diphosphate molecules, including chlorinated analogues, molecules having a hydrophilic moiety in their alkyl chain, or cyclic diphosphate molecules in previous *in vitro* studies,^216–219^ and were reviewed recently.^220^ Because these irregular and modified prenyl diphosphates differ structurally from their native counterparts, they could be used to create orthogonal pathways to terpenoids in *E. coli*, or expand the end-product range.

## 5 Bacterial terpenoid synthases and modifying enzymes

Prenyl diphosphates are used by TPSs to make terpenoid backbones, which subsequently can be modified by additional enzymes to make functionalised structures. TPSs and modifying enzymes were first mostly elucidated in plants and therefore most studies in *E. coli* and most of the highest titre reports use genes of plant origin (Table S1†). However, eukaryotic proteins expressed in *E. coli* can fold incorrectly and form inclusion bodies, and in general are not successfully expressed in bacteria,^221^ even after codon optimisation and removal of eukaryotic elements such as N-terminal signal peptides. A lack of eukaryote-specific post-translational modifications can also lead to less than optimal catalytic activities^222^ or different product profiles, which might impact terpenoid titres. Expressing soluble protein is particularly important for TPSs because they often catalyse the bottleneck step in terpenoid production, especially after precursor supply optimisation.^46,172,223^ Also, toxic intermediates like FPP can accumulate without efficient uptake by TPSs.^46^ Indeed, few of the tested plant TPSs are sufficiently active to support high-titre production. For example, only 20 of a group of 37 plant monoterpenoid synthases tested together produced the expected monoterpenoid in *E. coli* and only approximately a quarter of these had sufficiently high titres to enable subsequent optimisation.^224^

In contrast, bacterial enzymes are more similar to *E. coli* proteins, and therefore more likely to be highly expressed, soluble, and catalytically active. In fact, the highest titres obtained in *E. coli* with bacterial TPSs are comparable or higher than those obtained with the corresponding plant TPSs. For example, bacterial *Streptomyces clavuligerus* 1,8-cineole synthase (CinS) produces 23.4 mg L^−1^ 1,8-cineole, comparable to 23.6 mg L^−1^ 1,8-cineole produced with plant *Salvia fruticosa* CinS, and higher than 9.4 mg L^−1^ 1,8-cineole produced with plant *A. thaliana* CinS and 3.6 mg L^−1^ 1,8-cineole produced with plant *Citrus unshiu* CinS in a direct comparison.^225,226^ In this case, the bacterial enzyme also produced almost exclusively 1,8-cineole which is not the case with plant enzymes (96% *vs*. 42–67%). In an independent study, the same *S. clavuligerus* CinS produces 228 mg L^−1^ 1,8-cineole, consistently higher but comparable to 200 mg L^−1^ using the fungal *Hypoxylon* sp. CinS. Further optimisation allows production of 653 mg L^−1^ 1,8-cineole using this bacterial CinS,^165^ the highest 1,8-cineole titre in *E. coli* reported so far. Additionally, bacterial *S. clavuligerus* linalool synthase (LinS) allowed accumulation to 72.7 mg L^−1^ linalool, 300 times the 0.26 mg L^−1^ linalool titre produced by plant *Artemisia annua* LinS.^225,226^ When comparing the highest reported titres, bacterial LinS led to more linalool accumulation than plant LinS: 1.03 g L^−1^ linalool was produced with bacterial *S. clavuligerus* LinS,^159^ whereas 601.2 mg L^−1^ linalool was produced with a fungal LinS,^227^ 505 mg L^−1^ linalool with plant *Mentha citrata* LinS^165^ and approximately 85 mg L^−1^ linalool with plant *Clarkia breweri* LinS.^228^ Thus, whereas several eukaryotic monoterpenoid synthases were screened to find high producers in *E. coli*, the only two identified bacterial monoterpenoid synthases produced higher or comparable titres to the best eukaryotic synthases.

Most of the TPS diversity comes from plant enzymes, but for a few terpenoids, only bacterial TPSs have been found, which highlights the value of bacterial TPSs despite the difficulty in identifying them. For example, *Streptomyces griseus* (+)-caryolan-1-ol synthase makes up to 406 mg L^−1^ sesquiterpenoids in *E. coli* including 100 mg L^−1^ caryophyllene and 10 mg L^−1^ caryolan-1-ol (449 mg L^−1^ total terpenoid).^229^ A bacterium such as *E. coli* might be the preferred chassis for production of these and other terpenoids made by bacterial TPSs. For example, the bacterial *S. clavuligerus* LinS that produced one of the highest linalool titres in *E. coli* produces none in *S. cerevisiae*, even though linalool can be produced in *S. cerevisiae* with plant *M. citrata* LinS.^230^ Also, bacterial *Streptomyces coelicor epi*-isozizane synthase and *Streptomyces* UC5319 pentalenene synthase produced 728 mg L^−1^*epi*-isozizaene and 780 mg L^−1^ pentalenene in *E. coli*, respectively, whereas 344 mg L^−1^ pentalenene was obtained in *S. cerevisiae*, although less titre optimisation was applied to the latter.^231^

Subsequent enzymes functionalise the terpenoid backbones made by TPSs and increase terpenoid diversity, creating additional molecules with biological activities of interest. Oxidations are usually catalysed by cytochromes P450 (CYPs) and eukaryotic CYPs are more abundant. CYPs are membrane proteins and so to enhance expression in *E. coli* and obtain high terpenoid titres, the N-terminal domain that contains a signal peptide for membrane insertion is usually modified to direct the protein to the plasma membrane, or truncated to direct to the cytosol.^232,233^ Thus, eukaryotic CYPs are successfully used for high-titre terpenoid production in *E. coli*. For example, plant *Artemisia annua* CYP71AV1 was used to make 105 mg L^−1^ artemisinic acid, which can be converted to the antimalarial artemisinin,^174^ and the plant *Taxus brevifolia* CYP taxadiene 5α-hydroxylase was used to make 58 mg L^−1^ taxadien-5α-ol.^40^ However, even following N-terminal domain engineering, eukaryotic CYPs are poorly expressed and have low activity in *E. coli* as eukaryotic CYPs are usually bound to the ER.^174^ The recent focus on bacterial terpenoid pathways is also identifying bacterial CYPs that have been used for terpenoid production in *E. coli*.^234^ Notable examples include using *Bacillus megaterium* CYP102A1 (BM3) engineered to change substrate specificity to convert amorphadiene to 250 mg L^−1^ artemisinic-11*S*,12-epoxide, which can in turn be converted to artemisinin,^173^ and *Mycobacterium* HXN-1500 CYP153A6 to convert limonene to 105 mg L^−1^ perillyl alcohol.^138^ To reach high terpenoid titres, CYPs require reducing partners, which work well at a ratio lower than 1 : 1. It is not a trivial exercise to determine which partners will work well with specific CYPs so several are often tested.^235,236^

There are few other examples of attempts to optimise titres using bacterial TPSs and modifying enzymes in *E. coli* (Table S1†). However, several bacterial TPSs, CYPs and reducing partners have been expressed in *E. coli* to characterise product profiles,^236–241^ which further suggests that bacterial terpenoid biosynthesis genes express well in *E. coli.* Titres might be improved further by optimising precursor pathways and performing protein engineering studies.

One of the challenges of identifying bacterial TPSs and modifying enzymes is that pathways for natural products biosynthesis are often silent under standard laboratory culture conditions. However, the advent of inexpensive whole-genome sequencing has made thousands of bacterial genomes available for mining of terpenoid pathways and new TPSs. Bacterial TPSs have been elusive because of low whole sequence similarity with eukaryotic TPSs. Recently, Hidden Markov Models, which are trained with known sequences to create profiles that might encode functionality, were used to identify candidate bacterial TPS.^242–244^ After several iterations, this approach suggested as many as 600 candidates (in addition to the almost ubiquitous geosmin or 2-methylisoborneol synthases) in the thousands of bacterial genomic sequences currently available, mostly in the *Streptomyces* genus.^245^ Experimental validation has confirmed the activity of over 70 bacterial TPSs for two monoterpenoids (1,8-cineole and linalool), over 40 sesquiterpenoids and over 30 diterpenoids.^240,245–247^ Therefore, there are a growing number of bacterial TPS candidates that could be used for terpenoid production.

With the demonstrated benefits of using bacterial TPSs and modifying enzymes, identification of new bacterial terpenoids and the characterisation of associated terpenoid pathways among the thousands of available genomic sequences is attractive. As pathways are often silent, different culturing conditions might need to be used to induce genes and identify compounds.^248^ Genes that encode natural product biosynthetic pathways are often clustered in genomes,^249^ and this is also true for the terpenoids, where TPS genes are physically close to genes for CYPs and other modifying enzymes. Therefore, identification of biosynthetic gene clusters is useful in genome mining.^250^ The bacterial genomes database “Antibiotics and Secondary Metabolites Analysis Shell” counts over 5000 putative terpenoid biosynthetic gene clusters.^251^ So far, over 70 clusters have been characterised and deposited in the Minimum Information about a Biosynthetic Gene cluster database with nucleotide sequences and product descriptions.^252^

The challenge is to characterise the large and growing number of remaining cryptic clusters. Natural product pathways can be characterised by laboriously expressing single genes or assembling putative pathways, but recent methodologies allow expression of whole gene clusters in *E. coli*, *Streptomyces* or other bacterial strains to identify products,^253^ and manipulation of native genomes to activate silent clusters.^254^ Additionally, omics approaches are also being used. For example, genomics are correlated with metabolomics in groups of closely related species with different terpenoid profiles to match metabolites to conserved gene clusters.^255^ Many of these approaches can be accelerated using high-throughput automated assembly, screening and MS analysis.^256,257^ Consequently, new bacterial terpenoid biosynthetic pathways will be identified and utilised in future high-titre production in *E. coli* or other bacterial hosts.

## 6 Handling toxicity

### 6.1 Mechanisms of toxicity

Production of several terpenoids seems to reach a maximum limit despite additional attempts to increase titres through optimising biosynthetic pathways. This limitation might be imposed by end-product toxicity. Terpenoid toxicity varies between reports perhaps due to the different conditions used, and few reports analyse multiple terpenoids under the same conditions. That said, in general, several terpenoids, such as isoprenol, limonene, linalool, sabinene, bisabolol, farnesene, and sclareol inhibit *E. coli* growth at g L^−1^ levels (Table 2), which are levels similar to the highest titres produced (Table 1).

**Table 2:** Selected reports of exogenous terpenoid toxicity to *E. coli* under typical production conditions^a^

Terpenoids and derivatives	Minimum concentration that causes growth reduction but not complete inhibition (g L^−1^)	MIC (>indicates that total inhibition was not reached at that concentration) (g L^−1^)	*T* (°C)	Scale (mL)	Reference
**Hemiterpenoids**					
Isoprenol	1.5	2.4	37	∼1?	274

**Monoterpenoids**					
α−Pinene	4.29	>42.9	37	0.8	275
α−Pinene	0.429	1.28	37	5	281
1,8-Cineole			37	0.1	321
Geraniol	1.78	8.89	30	5	273
Geraniol	0.222	0.444	37	0.8	275
Geraniol	0.075	0.300	37	1	163
-Geranyl acetate	4.58	>45.8	37	0.8	275
-Geranyl acetate	0.5		37	1	163
Myrcene	>7.94	>7.94	30	5	273
Sabinene	1	>5	31	50	161
Linalool		1	37	0.2	159
Limonene	1.05	42.05	30	0.1?	264
Limonene	>25.23	>25.23	30	0.15	263
Limonene	3.36	8.41	37	5	281
Limonene	0.05	0.21	37	0.8	275
-Perillyl alcohol		4.79	30	0.15	263

**Sesquiterpenoids**					
α−Bisabolene	>180	>180	37	1	322
Bisabolol	>27.6	>27.6	30	0.15	263
(−)-α−Bisabolol	5	>5			309
γ-Bisabolene	>27	>27	30	0.15	263
Farnesol	>26.61	>26.61	30	0.15	263
α−Farnesene	>24.39	>24.39	30	0.15	263

**Diterpenoids**					
*Cis*-Abienol	>2	>2	37	10	183
Cembratriene-ol	>2	>2	37	2	45
Sclareol	>2	>2	37	10	185

a Where concentrations were expressed as “%”, “% v/v” was assumed for liquid terpenoids and g L^−1^ were calculated using density.

**Fig. 6:**
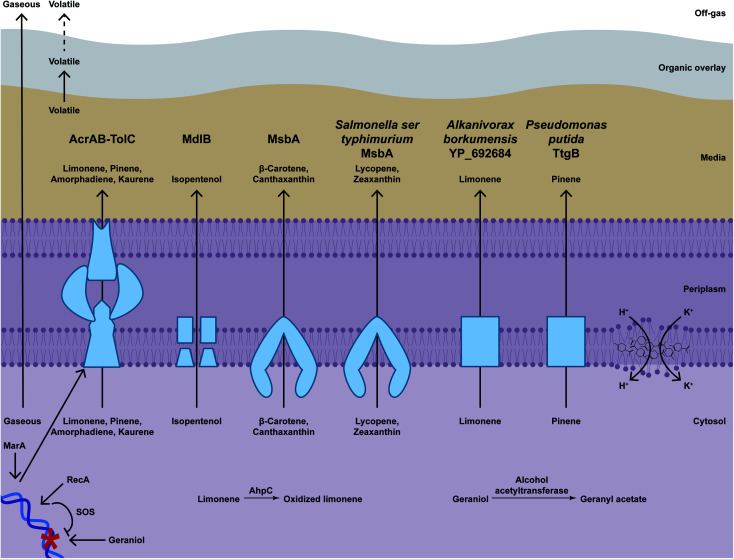
Mechanisms of terpenoid toxicity and resistance. Terpenoids can accumulate in membranes (indicated with limonene structures) and allow leakage. Terpenoids have other toxic effects. For example, geraniol can damage DNA (indicated with blue double helix), which can be repaired by RecA and the SOS repair response, and limonene can be oxidised to a more toxic derivative *in vivo*. Gaseous terpenoids can evaporate to the off-gas. Volatile terpenoids can be captured by an organic overlay. Efflux pumps export terpenoids (indicated with blue boxes) and can be induced by MarA. Bioderivatisation converts toxic terpenoids into less toxic derivatives. For example, geraniol can be converted to geranyl acetate.

In general, terpenoids are hydrophobic hydrocarbons and therefore can accumulate in biological membranes and increase membrane permeability, thus disrupting vital biological processes (*e.g.* by allowing proton leakage and dissipating the proton motive force needed for energy production; Fig. 6).^258,259^ A commonly used metric for hydrophobicity is the logarithm of the partition coefficient between octanol and water (log *P*).^260^ Hydrocarbons with log *P* between 1 and 4 partition easily into membranes and therefore can be toxic;^258^ above log *P* 4 water solubility also determines toxicity.^260^

Hydrophobicity might also alter secretion times. For example, even when efflux pumps are induced to export terpenoids, zeaxanthin accumulates in the broth at 72 h post-induction, whereas the more hydrophobic canthaxantin peaks at 96 h, and the more hydrophobic β-carotene takes 120–144 h to accumulate extracellularly; lycopene requires outer membrane removal through spheroplast formation to detect extracellular levels.^261^ Consistently, carotenoids accumulate in the inner membrane instead of in the media because of low water solubility in *E. coli*.^261^

Some structural features can also determine toxicity. In general, hydroxyl groups and double bonds can increase toxicity.^262^ Thus, some terpenoids can be toxic at lower concentrations than other similarly hydrophobic terpenoids. For example, *E. coli* grows as well with 25.23 g L^−1^ exogenously added limonene as a non-supplemented control, so limonene itself might not be toxic. But oxidised forms of limonene such as terpineol and perillyl alcohol inhibit growth almost completely at concentrations of 4.67 g L^−1^ and 4.79 g L^−1^, respectively.^263^ Also, limonene is oxidised *in vivo* to a hydroperoxide that might cause oxidative damage, as evidenced by the finding that an endogenous alkyl hydroperoxidase AhpC (L177Q) variant alleviates toxicity.^264^ Another example is that of geraniol; in one report, 75 mg L^−1^ exogenous geraniol reduces growth rate and 300 mg L^−1^ inhibits growth entirely,^163^ whereas similar terpenoids are toxic in the g L^−1^ range (Table 2). This additional toxicity might be due to DNA damage; *recA*, which encodes a protein that activates SOS box genes for DNA repair, confers resistance to geraniol and, consistently, *ΔrecA* strains are more geraniol sensitive than wild-type counterparts.^265^ Therefore, toxicity can be hard to predict based on terpenoid structure and rather is assessed empirically.

Terpenoid production in *E. coli* therefore needs to address arguably the last step in the biosynthetic pathway *i.e.* removal of the end-product from the cell, and even from the media, to handle end-product toxicity, as well as to achieve efficient recovery and purification from the production broth. Of note, the concentration of exogenously added terpenoids that can be tolerated might be higher than the maximum titres that cells can produce, because the intracellular concentration is expected to be higher when the cell produces the terpenoid than when it is added externally.

### 6.2 Physical extraction

Physically removing terpenoids from the media reduces the exposure of the cells to the end product. Some terpenoids with high volatility simply evaporate. For example, isoprene is gaseous and volatilises from the cultivation medium, which enables production to 60 g L^−1^ isoprene in *E. coli*.^76^ Other terpenoids might form salts that precipitate out of solution. For example, artemisinic acid is known to accumulate on the walls of cultivation flasks and therefore is produced to high titres in *S. cerevisiae*.^266^

While volatility prevents toxicity, it also leads to product loss and titre underestimation. Therefore, condensers and gas-stripping are often used for product recovery from the culture off-gas. Also, a hydrophobic overlay of an organic solvent chosen to maximise terpenoid solubilisation can be directly applied to the culture medium to create a two-phase fermentation system (Fig. 6). For example, titres of the volatile amorphadiene increase from 24 mg L^−1^ to 281 mg L^−1^ when using a condenser to trap volatiles in the off-gas and dodecane as an organic overlay; by further improving culturing conditions, titres reach 0.5 g L^−1^ amorphadiene with this recovery method.^267^

Organic overlays also help handle toxicity of less volatile terpenoids, perhaps by displacing the equilibrium from the media and therefore reducing exposure of cells to the end product. Many examples of the highest titres achieved so far report using overlays (Tables 1 and S1†). Solid adsorbents similarly sequester terpenoids in culture media and increase titres (Tables 1 and S1†).

### 6.3 Secretion

Microbes can be engineered to enhance hydrocarbon resistance, and thus also increase production titres. One of the most common approaches is to increase secretion by inducing or overexpressing efflux pumps, which have broad substrate specificity and export metabolites that might be toxic (Fig. 6).^268^

The multiprotein AcrAB-TolC is the main *E. coli* pump that increases terpenoid tolerance and production titres. AcrAB-TolC is composed of AcrB, an ATP-binding cassette (ABC) transporter in the inner membrane, TolC, an outer membrane channel, and AcrA, an accessory protein in the periplasmic space that mediates interaction between AcrB and TolC to export the substrate through the two membranes.^269^ Overexpressing *tolC* increases amorphadiene titres from approximately 45 mg L^−1^ to approximately 160 mg L^−1^.^270^ However, overexpressing *acrAB* has modest, if any, effect on pinene titres, which range from 7.3 to 8.1 mg L^−1^.^271^ Also, overexpressing *tolC* is reported to increase amorphadiene titres from 250 mg L^−1^ to approximately 350 mg L^−1^ but kaurene production is perhaps not significantly affected (from approximately 17 mg L^−1^ to approximately 21 mg L^−1^). Combining efflux pump components can also further increase titres. Overexpressing *acrB* and two copies of *tolC* increases titres to 404.8 mg L^−1^ amorphadiene and overexpressing *acrA*, *acrB* and *tolC* increases titres to 31.76 mg L^−1^ kaurene.^187^ AcrAB-TolC is positively regulated by MarA, a global transcriptional factor that induces efflux pumps.^272^ Overexpression of *marA* allows growth on 8.9 g L^−1^ geraniol, which otherwise completely inhibits growth, and reduces intracellular geraniol levels from 12.9 μg mg^−1^ to 6.3 μg mg^−1^, suggesting that efflux pumps might act by reducing intracellular terpenoid concentrations.^273^

Other *E. coli* pumps also increase terpenoid titres. For example, overexpressing *mdlB*, which encodes a putative ABC transporter, increases isopentenol titres by 12% to 931 mg L^−1^.^274^ Also, overexpressing *msbA*, which encodes an ABC transporter that transports the lipid A-core moiety of lipopolysaccharide from the inner leaflet to the outer leaflet of the inner membrane, increases canthaxantin 4.4-fold from approximately 39 μg L^−1^ to approximately 170 μg L^−1^ and β-carotene also 4.4-fold to approximately 250 μg L^−1^.^261^

Expressing pumps from other organisms also improves tolerance and production. For example, expressing *Alcanivorax borkumensis* YP_692684 modestly increases limonene titres from approximately 35 mg L^−1^ to approximately 55 mg L^−1^.^275^ In addition, expressing *Salmonella enterica* serovar *typhimurium msbA* with a mutation that results in a I89T change increases zeaxanthin 2.4-fold from approximately 100 μg L^−1^ to approximately 249 μg L^−1^ and secreted lycopene 4.3-fold from approximately 48 μg L^−1^ to approximately 210 μg L^−1^.^261^ Expressing *Pseudomomas putida ttgB* however has very modest (if any) effects on titres (from 8.1 to 9.1 mg L^−1^ pinene).^271^

Efflux pumps differ in terpenoid specificity. For example, a combination of efflux pumps that is optimal for increasing amorphadiene titres is different than the optimal combination for increasing kaurene titres.^187^ Also, when testing a group of pumps, StMbsA increases zeaxanthin and lycopene titres the most, whereas EcMbsA increases canthaxanthin the most.^261^ Similarly, several pumps that increase amorphadiene production do not increase lycopene production.^270^

Therefore, the use of efflux pumps seems to provide some advantage, although the effects in titre can be modest. This suggests that terpenoid production is affected by terpenoid toxicity or intracellular accumulation, but the effects so far are limited, providing about 4-fold titre increase at most. The limitation might be because overexpressing pumps also inhibit bacterial growth.^276^ Export could be improved through protein engineering to increase specificity and/or activity, particularly with AcrB, which recognises substrates in the cytosol and the inner membrane. Importantly, use of inducing pumps have not yet been reported in most of the higher titre producing systems where they would be expected to be the most beneficial.

### 6.4 Membrane engineering

Because terpenoids accumulate in membranes, extending membranes can increase the storage capacity for terpenoids and lead to higher terpenoid production while still allowing cellular functions. There are several *E. coli* genes that ‘bend’ membranes when overexpressed, causing invaginations and increasing levels of membrane constituents. For example, overexpressing *tsr*, which encodes a methyl-accepting chemotaxis protein, promotes the formation of membrane invaginations and extensions, and increases squalene titres 2.3-fold from 272 to 612 mg L^−1^, the highest squalene titre reported to date.^277^ Similarly, overexpressing *almgs*, which encodes monoglucosyldiacylglycerol synthase, causes membrane extension and improves β-carotene titres 1.5-fold from 24.7 mg L^−1^ to 36.9 mg L^−1^. Also, overexpressing two genes involved in diglyceride-3-phosphate biosynthesis increases glycerophospholipids (membrane components) and β-carotene titres 1.3-fold (to 31.3 mg L^−1^). Overexpressing these last three genes together leads to a further synergistic improvement, increasing titres 4.2-fold relative to the parental strain to 103.5 mg L^−1^ β-carotene. In a separate β-carotene hyperproducing strain, this membrane engineering leads to a 1.4-fold titre increase from 196.3 mg L^−1^ to 268.1 mg L^−1^ β-carotene.^278^ These early results show promise for further rounds of membrane engineering, combining membrane-altering factors to increase storage capacity and terpenoid titres while preserving cell integrity and growth rates.

### 6.5 Excretion

An alternative way of removing terpenoids from cells is to stimulate excretion. *E. coli* excretes outer membrane vesicles when deleting *tolA* or *tolR*, which encode components of the Tol complex that maintains outer membrane integrity, or *nlpI*, which encodes an outer membrane-bound protein that might contribute to protein complex formation. Thus, deleting *nlpI* together with either *tolA* or *tolR* increases β-carotene excretion and total production. Additionally, overexpressing genes in the biosynthetic pathway of phosphatidylethanolamine, the main phospholipid in *E. coli* membranes, to supply additional membrane for the excretion further increases β-carotene production 71-fold relative to the parent strain (final production of 10.7 mg g^−1^ DCW β-carotene). Carrying out similar changes in a β-carotene hyperproducing strain leads to a similar 24-fold production increase relative to the parent strain (reaching 44.8 mg g^−1^ DCW β-carotene^279^).

### 6.6 Adaptive laboratory evolution

Because toxicity is multi-causal and can be specific to each terpenoid, adaptive laboratory evolution experiments which address toxicity in a more general fashion are a good strategy to increase terpenoid titres. Adaptive laboratory evolution on *E. coli* grown on media supplemented with pinene increases tolerance from 5 to 20 g L^−1^, and increases titres by 31% to 7.3 mg L^−1^ pinene.^271^ Also, evolving *E. coli* to grow on increasing sabinene concentrations from 3 g L^−1^, below the initial point of no growth at 3.5 g L^−1^, to 12 g L^−1^ generates a strain with 8-fold increased production (from 22.8 mg L^−1^ to 191.8 mg L^−1^). In this strain, a large number of genes have mutations or altered expression levels. Three genes in particular contribute to sabinene tolerance: *ycbK*, *scpA*, and *ygiZ*. *ycbK* is a gene of unknown function that belongs to the defective Lambda prophage 12 family with roles in biofilm formation, stress response and cell wall maintenance. *scpA* encodes methylmalonyl-CoA mutase and affects central metabolism. *ygiZ* encodes an internal membrane protein of unknown function and is induced by the BglJ-RcsB transcriptional activator that regulates motility, biofilm formation and stress responses. Overexpressing *ycbK* or *scpA* increases sabinene titres approximately 2-fold, but overexpressing *ygiZ* decreases sabinene titres.^280^ This work illustrates the multi-causality of toxicity and how engineering tolerance might require a multi-pronged approach involving several relevant cellular processes simultaneously and mechanisms we still do not fully understand.

Similarly, other genes increase terpenoid resistance and production titres but by poorly defined mechanisms. For example, several genes are upregulated in response to *E. coli* cultivation with isopentenol. Six of these genes involved in oxidative stress response (*fpr*), general stress response (*metR*, *yqhD*, and *gidB*), heat shock-related response (*ibpA*) and transport (*mdlB*) increase isopentenol titres when overexpressed. Overexpressing *metR*, which encodes a DNA-binding transcriptional activator, leads to the highest improvement (55%, from 838 mg L^−1^ to 1290 mg L^−1^ isopentenol^274^). Like efflux pumps, genes granting tolerance and titre increases might be terpenoid specific. For example, overexpressing *Marinobacter aquaeolei* VT8 *yceI* improves resistance to pinene and terpinolene, but not to limonene or terpinene.^281^

### 6.7 Bioderivatisation

Bioderivatisation consists of converting the target terpenoid to a less toxic derivative inside the cell to enable accumulation to high titres and subsequently reconverting the derivative to the original target terpenoid.^282^ This approach was partially applied to the particularly toxic geraniol, which reduces growth rate at 75 mg L^−1^ and fully inhibits growth at 300 mg L^−1^.^163^ In contrast, geranyl acetate, an esterified derivative of geraniol, reduces final optical density (OD) by 40% at 4.6 g L^−1^ but then no further reduces OD up to 45.8 g L^−1^ (probably because it forms a second phase at concentrations higher than its aqueous solubility,^275^ which is lower than that of geraniol^163^). Therefore, expressing the gene encoding *Rosa hybrida* alcohol acetyltransferase in *E. coli* to catalyse intracellular esterification of geraniol increases titres from 35 mg L^−1^ geraniol to 375 mg L^−1^ geranyl acetate, which represents a more than 8-fold molar increase. By further optimising conditions, 4.8 g L^−1^ geranyl acetate was produced,^163^ over 2-fold higher than the previously reported maximum of 2 g L^−1^ geraniol.^162,170^ Esterification also prevents spontaneous conversion to toxic geraniol derivatives. Conversion of geranyl acetate back to geraniol however was not discussed in this report. To that end, an endogenous *E. coli* acetylesterase hydrolyses geranyl acetate to geraniol.^162^ So far it has been used to increase geraniol levels by preventing spontaneous conversion to geranyl acetate, but has not yet been utilised in a bioderivatisation strategy of the type described above. Expressing this acetylesterase with a secretion tag that allows reconversion outside the cell could potentially enable recovery of geraniol when using bioderivatisation approaches.

In summary, several approaches have been used separately in attempts to mitigate product toxicity. In many cases, the benefits are modest but a combination of approaches could in principle give rise to synergistic effects. Available evidence suggests that further combinatorial analysis of these types of approaches is warranted.

## 7 Conclusions and future perspectives

**Fig. 7:**
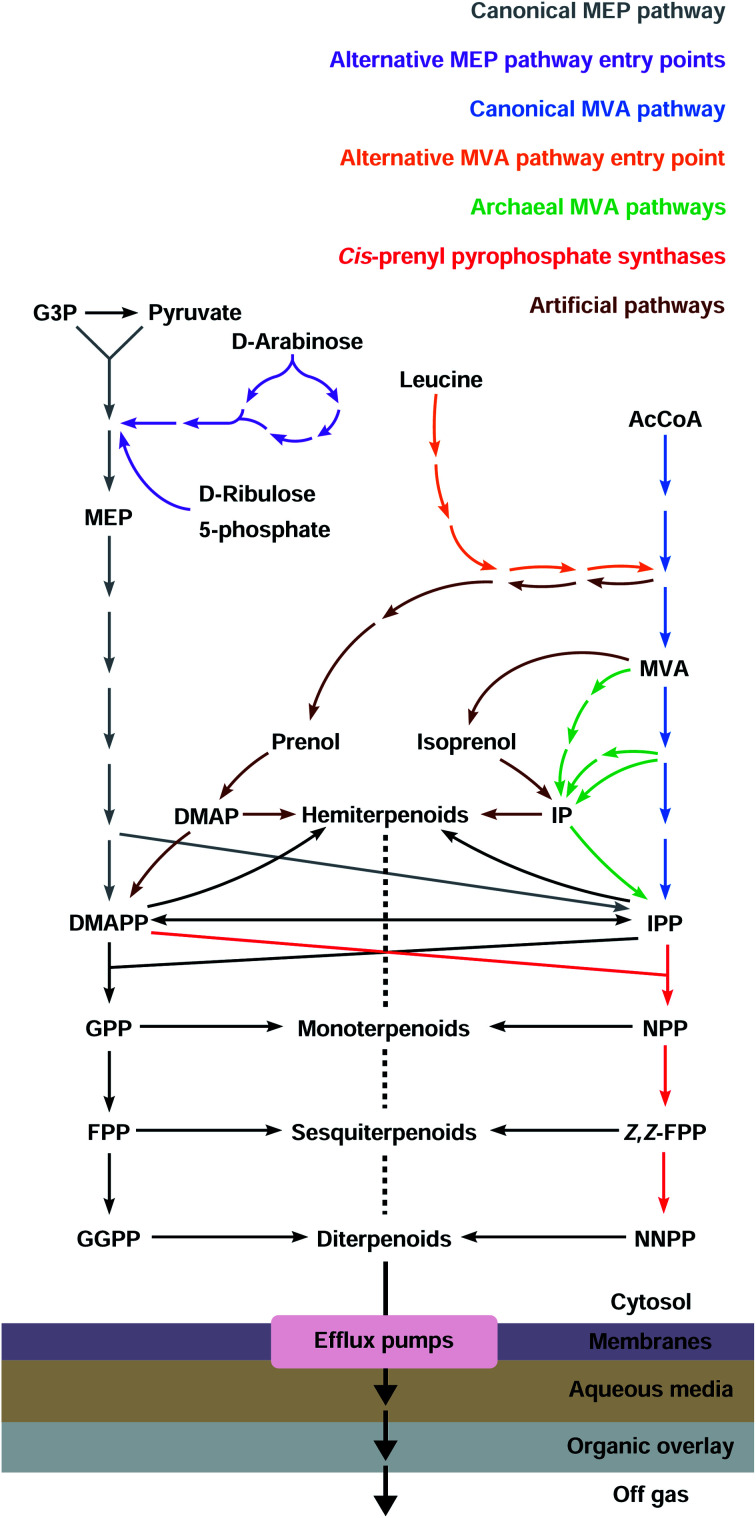
Summary of terpenoid pathways. The MEP pathway is native to *E. coli*. The MVA pathway has been inserted in *E. coli* and led to higher terpenoid titres. In addition, alternative pathways have been tested in *E. coli* or could provide benefits in terpenoid production in *E. coli*.

The use of alternative and artificial biosynthetic pathways is now demonstrated for the production of terpenoids in metabolic engineering programs (Fig. 7). The ever-increasing efforts to understand the rich diversity of terpenoid biosynthetic chemistry in nature, particularly ongoing searches for new bacterial TPSs and modifying enzymes, and pathways to their synthesis, will provide new opportunities for the future microbial production of terpenoids. Where natural pathways are not currently available, retrosynthesis tools will be used to suggest artificial biosynthetic pathway designs and enable searches for suitable candidate enzymes.^283,284^ The studies described in this review have helped to validate the use of enzymes within a pathway context and provide useful frameworks for further optimisation to reach high-titre terpenoid production. It goes without saying that enzyme engineering, including both rational design and directed evolution, will continue to contribute to this field. This might involve the engineering of enzymes that are more resistant to feedback control, or enzymes with improved catalytic efficiency, or new specificities towards desired target activities/products.^77,137,168,173,186,198,257,285,286^ The use of alternative and artificial pathways with this ever-expanding enzyme resource and pathway engineering tools should extend current capabilities, thereby allowing high-titre production of a wide range of terpenoids in *E. coli* and other microbial species.

Extensive studies of the MEP and the MVA pathways have demonstrated that balancing is required to (i) avoid the accumulation of toxic intermediates, (ii) overcome the limiting effects of regulation and (iii) optimise metabolic flux. Complete understanding of canonical pathway regulation and how accumulation of pathway intermediates limits flux remain to be elucidated. This is necessary to inform pathway balancing realised through engineering and modelling, and will likely involve combinatorial approaches that address a range of issues of the type identified in this review.

High-throughput methodologies that harness automation and miniaturisation to construct and assess the capabilities of newly engineered strains will become increasingly important to assemble new genetic constructs and test terpenoid production. Automated Design-Build-Test-Learn platforms that are embedded in biofoundry approaches for microbial strain engineering will provide the basis for such work. This will enable rapid exploration of DNA parts (*e.g.* promoters, ribosome binding sites)^287–289^ to identify improved pathways and target compound production,^41,256,290^ with an increased emphasis on the use of data-driven predictive engineering.^289,291,292^ Establishing high-throughput screening protocols for terpenoids beyond available low sensitivity and non-specific methods will be important.^293^ Currently, GC-MS is widely used in the field but suffers from low throughput. Amongst others, developments in high-throughput GC-QTOF, which measures terpenoids from the small volumes of multi-well plates, is potentially an attractive approach.^257^

Balancing central metabolism precursors, replacing the carbon source, modifying the energy and reducing power balance in the cell, and other approaches will also need to be explored alongside the engineering of new biosynthetic pathways. Redirecting carbon flux and balancing precursor levels are known to increase terpenoid levels,^294–298^ as is enhanced availability of essential cofactors such as ATP and NADPH.^299,300^ Other compounds of value are also produced by pathways that require the same metabolic precursors used for terpenoid production,^301^ with opportunities for learning across different product types. End-product toxicity remains as a major challenge for high-titre terpenoid production. This will require new solutions from microbial strain engineering and also biochemical engineering (*e.g. in situ* product removal in continuous culture). Ultimately, production from renewable feedstocks is required. Limited examples are available, such as the production of pinene from pre-treated switchgrass^302^ and hydrolysed macroalgae,^303^ and isopentenol produced from pre-treated and hydrolysed lignocellulose.^304^ Production may need also to migrate into industrial production hosts to take advantage of agricultural or industrial feedstock waste streams, or carbon dioxide.^305,306^ There is clearly much to do. This review will hopefully define some of the key challenges and strategies to achieve high level production of terpenoids in *E. coli*. The hope is that through this expanding knowledge base large-scale production of terpenoids using microbial cell factories will become a reality.

## 8 Abbreviations

AACT: Acetoacetyl-CoA thiolase
ABC: ATP binding cassette
AcAcCoA: Acetoacetyl-CoA
AcCoA: Acetyl-CoA
AgGPPS: *Abies grandis* geranyl diphosphate synthase
AMPD: *trans*-Anhydromevalonate 5-phosphate decarboxylase
AR: Aldehyde reductase
BMD: Mevalonate biphosphate decarboxylase
CPD-ME: 4-Diphophocytidyl-2-*C*-methyl-d-erythritol
CDP-MEP: 4-Diphophocytidyl-2-*C*-methyl-d-erythritol 2-phosphate
CinS: 1,8-Cineole synthase
CMK: 4-Diphophocytidyl-2-*C*-methyl-d-erythritol kinase
CMS: 4-Diphophocytidyl-2-*C*-methyl-d-erythritol synthase
CTP: Cytidine 5′-triphosphate
CYP: Cytochrome P450
DHAP: Dihydroxyacetone phosphate
DMAP: Dimethylallyl phosphate
DMAPP: Dimethylallyl diphosphate
DMD: Mevalonate diphosphate decarboxylase
DX: 1-Deoxy-d-xylulose
DXP: 1-Deoxy-d-xylulose 5-phosphate
DXR: 1-Deoxy-d-xylulose 5-phosphate reductoisomerase
DXS: 1-deoxy-d-xylulose 5-phosphate synthase
FPP: Farnesyl diphosphate
FPPS: Farnesyl diphosphate synthase
FSA: Fructose-6-phosphate aldolase
G3P: d-Glyceraldehyde 3-phosphate
GA: Glycolaldehyde
GGPP: Geranylgeranyl diphosphate
GGPPS: Geranylgeranyl diphosphate synthase
GPP: Geranyl diphosphate
GPPS: Geranyl diphosphate synthase
HA: Hydroxyacetone
HDR: 4-Hydroxy-3-methyl-butenyl diphosphate reductase
HDS: 4-Hydroxy-3-methyl-butenyl diphosphate synthase
HMBPP: 4-Hydroxy-3-methyl-butenyl diphosphate
HMG-CoA: 3-Hydroxy-3-methylglutaryl-CoA
HMGR: 3-Hydroxy-3-methylglutaryl-CoA reductase
HMGS: 3-Hydroxy-3-methylglutaryl-CoA synthase
IDI: Isopentenyl diphosphate delta-isomerase
IP: Isopentenyl phosphate
IPK: Isopentenyl phosphate kinase
IPP: Isopentenyl diphosphate
LinS: Linalool synthase
M3K: Mevalonate 3-kinase
M3P: Mevalonate 3-phosphate
M3P5K: Mevalonate 3-phosphate 5-kinase
M3P5P: Mevalonate 3,5-biphosphate
M5K: Mevalonate 5-kinase
M5P: Mevalonate 5-phosphate
MB: 3-Methyl-2-butenal
MB-CoA: 3-Methyl-2-butenoyl-CoA
MCS: 2-*C*-Methyl-d-erythritol 2,4-cyclodiphosphate synthase
MEcPP: 2-*C*-Methyl-d-erythritol 2,4-cyclodiphosphate
MEP: 2-*C*-Methyl-d-erythritol 4-phosphate
MG: Methylglyoxal
MG-CoA: 3-Methylglutaconyl-CoA
MGS: Methylglyoxal synthase
MVA: Mevalonate
MVAPP: Mevalonate diphosphate
NNPP: Nerylneryl diphosphate
NNPPS: Nerylneryl diphosphate synthase
NPP: Neryl diphosphate
NPPS: Neryl diphosphate synthase
OD: Optical density
PMD: Mevalonate 5-phosphate decarboxylase
PMDh: Phosphomevalonate dehydratase
PMK: Phosphomevalonate kinase
PPPS: Prenyl diphosphate synthase
SlNPPS: *Solanum lycopersicum* neryl diphosphate synthase
tAHMP: *trans*-Anhydromevalonate 5-phosphate
TPS: Terpenoid synthase
XK: d-Xylulose kinase
zFPPS: *Z*,*Z*-Farnesyl diphosphate synthase

## 9 Author contributions

MAR: conceptualisation, visualisation, writing – original draft, writing – review & editing. CAF: conceptualisation, writing – original draft, writing – review & editing. NSS: conceptualisation, supervision, funding acquisition, writing – review & editing.

## 10 Conflicts of interest

There are no conflicts to declare.

## Supplementary Material

NP-039-D1NP00025J-s001

## References

[cit1] Buckingham J. , Dictionary of natural products, supplement 4, CRC press, 1997

[cit2] Tetali S. D. (2019). Planta.

[cit3] Clomburg J. M., Gonzalez R. (2010). Appl. Microbiol. Biotechnol..

[cit4] Zebec Z., Wilkes J., Jervis A. J., Scrutton N. S., Takano E., Breitling R. (2016). Curr. Opin. Chem. Biol..

[cit5] Niu F.-X., Lu Q., Bu Y.-F., Liu J.-Z. (2017). Synthetic and Systems Biotechnology.

[cit6] Moser S., Pichler H. (2019). Appl. Microbiol. Biotechnol..

[cit7] Kung S. H., Lund S., Murarka A., McPhee D., Paddon C. J. (2018). Front. Plant Sci..

[cit8] Meadows A. L., Hawkins K. M., Tsegaye Y., Antipov E., Kim Y., Raetz L., Dahl R. H., Tai A., Mahatdejkul-Meadows T., Xu L., Zhao L., Dasika M. S., Murarka A., Lenihan J., Eng D., Leng J. S., Liu C. L., Wenger J. W., Jiang H., Chao L., Westfall P., Lai J., Ganesan S., Jackson P., Mans R., Platt D., Reeves C. D., Saija P. R., Wichmann G., Holmes V. F., Benjamin K., Hill P. W., Gardner T. S., Tsong A. E. (2016). Nature.

[cit9] Wu W., Maravelias C. T. (2018). Biotechnol. Biofuels.

[cit10] Sun C., Theodoropoulos C., Scrutton N. S. (2020). Bioresour. Technol..

[cit11] Shukla V., Phulara S. C. (2021). Appl. Environ. Microbiol..

[cit12] Pandit A. V., Srinivasan S., Mahadevan R. (2017). Nat. Commun..

[cit13] Henry L. K., Gutensohn M., Thomas S. T., Noel J. P., Dudareva N. (2015). Proc. Natl. Acad. Sci. U. S. A..

[cit14] Thomas S. T., Louie G. V., Lubin J. W., Lundblad V., Noel J. P. (2019). ACS Chem. Biol..

[cit15] Lois L. M., Campos N., Putra S. R., Danielsen K., Rohmer M., Boronat A. (1998). Proc. Natl. Acad. Sci. U. S. A..

[cit16] Rohmer M., Seemann M., Horbach S., Bringer-Meyer S., Sahm H. (1996). J. Am. Chem. Soc..

[cit17] Kuzuyama T., Takahashi S., Watanabe H., Seto H. (1998). Tetrahedron Lett..

[cit18] Kuzuyama T., Takagi M., Kaneda K., Dairi T., Seto H. (2000). Tetrahedron Lett..

[cit19] Kuzuyama T., Takagi M., Kaneda K., Watanabe H., Dairi T., Seto H. (2000). Tetrahedron Lett..

[cit20] Takagi M., Kuzuyama T., Kaneda K., Watanabe H., Dairi T., Seto H. (2000). Tetrahedron Lett..

[cit21] Hecht S., Eisenreich W., Adam P., Amslinger S., Kis K., Bacher A., Arigoni D., Rohdich F. (2001). Proc. Natl. Acad. Sci. U. S. A..

[cit22] Kollas A.-K., Duin E. C., Eberl M., Altincicek B., Hintz M., Reichenberg A., Henschker D., Henne A., Steinbrecher I., Ostrovsky D. N. (2002). FEBS Lett..

[cit23] Rohdich F., Zepeck F., Adam P., Hecht S., Kaiser J., Laupitz R., Gräwert T., Amslinger S., Eisenreich W., Bacher A., Arigoni D. (2003). Proc. Natl. Acad. Sci. U. S. A..

[cit24] Rohdich F., Hecht S., Gärtner K., Adam P., Krieger C., Amslinger S., Arigoni D., Bacher A., Eisenreich W. (2002). Proc. Natl. Acad. Sci. U. S. A..

[cit25] Altincicek B., Duin E. C., Reichenberg A., Hedderich R., Kollas A.-K., Hintz M., Wagner S., Wiesner J., Beck E., Jomaa H. (2002). FEBS Lett..

[cit26] Hahn F. M., Hurlburt A. P., Poulter C. D. (1999). J. Bacteriol..

[cit27] Zhao L., Chang W. C., Xiao Y., Liu H. W., Liu P. (2013). Annu. Rev. Biochem..

[cit28] Banerjee A., Sharkey T. (2014). Nat. Prod. Rep..

[cit29] Banerjee A., Wu Y., Banerjee R., Li Y., Yan H., Sharkey T. D. (2013). J. Biol. Chem..

[cit30] Matthews P., Wurtzel d. E. T. (2000). Appl. Microbiol. Biotechnol..

[cit31] Zhao Y., Yang J., Qin B., Li Y., Sun Y., Su S., Xian M. (2011). Appl. Microbiol. Biotechnol..

[cit32] Kim S. W., Keasling J. D. (2001). Biotechnol. Bioeng..

[cit33] Li Y., Wang G. (2016). J. Appl. Microbiol..

[cit34] Li Q., Fan F., Gao X., Yang C., Bi C., Tang J., Liu T., Zhang X. (2017). Metab. Eng..

[cit35] Yuan L. Z., Rouviere P. E., Larossa R. A., Suh W. (2006). Metab. Eng..

[cit36] Lv X., Xu H., Yu H. (2013). Appl. Microbiol. Biotechnol..

[cit37] Du F.-L., Yu H.-L., Xu J.-H., Li C.-X. (2014). Bioresources and Bioprocessing.

[cit38] Bitok J. K., Meyers C. F. (2012). ACS Chem. Biol..

[cit39] Zhou K., Zou R., Stephanopoulos G., Too H. P. (2012). PLoS One.

[cit40] Ajikumar P. K., Xiao W.-H., Tyo K. E., Wang Y., Simeon F., Leonard E., Mucha O., Phon T. H., Pfeifer B., Stephanopoulos G. (2010). Science.

[cit41] Coussement P., Bauwens D., Maertens J., De Mey M. (2017). ACS Synth. Biol..

[cit42] Yang J., Guo L. (2014). Microb. Cell Fact..

[cit43] Rad S. A., Zahiri H. S., Noghabi K. A., Rajaei S., Heidari R., Mojallali L. (2012). World J. Microbiol. Biotechnol..

[cit44] Mischko W., Hirte M., Fuchs M., Mehlmer N., Brück T. B. (2018). Microb. Cell Fact..

[cit45] Mischko W., Hirte M., Roehrer S., Engelhardt H., Mehlmer N., Minceva M., Brück T. (2018). Green Chemistry.

[cit46] Martin V. J., Pitera D. J., Withers S. T., Newman J. D., Keasling J. D. (2003). Nat. Biotechnol..

[cit47] Zahiri H. S., Yoon S. H., Keasling J. D., Lee S. H., Won Kim S., Yoon S. C., Shin Y. C. (2006). Metab. Eng..

[cit48] Rudney H., Ferguson Jr J. J. (1957). J. Am. Chem. Soc..

[cit49] Chaykin S., Law J., Phillips A., Tchen T., Bloch K. (1958). Proc. Natl. Acad. Sci. U. S. A..

[cit50] Henning U., Möslein E., Lynen F. (1959). Arch. Biochem. Biophys..

[cit51] Tchen T. (1957). J. Am. Chem. Soc..

[cit52] Pitera D. J., Paddon C. J., Newman J. D., Keasling J. D. (2007). Metab. Eng..

[cit53] Liu C. L., Bi H. R., Bai Z., Fan L. H., Tan T. W. (2019). Appl. Microbiol. Biotechnol..

[cit54] Choi B. H., Kim J. H., Choi S. Y., Han S. J., Lee P. C. (2019). Bioresource Technology Reports.

[cit55] Kizer L., Pitera D. J., Pfleger B. F., Keasling J. D. (2008). Appl. Environ. Microbiol..

[cit56] George K. W., Chen A., Jain A., Batth T. S., Baidoo E. E., Wang G., Adams P. D., Petzold C. J., Keasling J. D., Lee T. S. (2014). Biotechnol. Bioeng..

[cit57] Ma S. M., Garcia D. E., Redding-Johanson A. M., Friedland G. D., Chan R., Batth T. S., Haliburton J. R., Chivian D., Keasling J. D., Petzold C. J. (2011). Metab. Eng..

[cit58] Alonso-Gutierrez J., Kim E.-M., Batth T. S., Cho N., Hu Q., Chan L. J. G., Petzold C. J., Hillson N. J., Adams P. D., Keasling J. D. (2015). Metab. Eng..

[cit59] Tanaka R. D., Lee L. Y., Schafer B. L., Kratunis V. J., Mohler W. A., Robinson G. W., Mosley S. T. (1990). Proc. Natl. Acad. Sci. U. S. A..

[cit60] Voynova N. E., Rios S. E., Miziorko H. M. (2004). J. Bacteriol..

[cit61] Yoon S. H., Lee Y. M., Kim J. E., Lee S. H., Lee J. H., Kim J. Y., Jung K. H., Shin Y. C., Keasling J. D., Kim S. W. (2006). Biotechnol. Bioeng..

[cit62] Woo H. M., Murray G. W., Batth T. S., Prasad N., Adams P. D., Keasling J. D., Petzold C. J., Lee T. S. (2013). Chem. Eng. Sci..

[cit63] Dorsey J. K., Porter J. W. (1968). J. Biol. Chem..

[cit64] Gray J., Kekwick R. (1972). Biochim. Biophys. Acta, Gen. Subj..

[cit65] Huang K.-x., Scott A., Bennett G. N. (1999). Protein Expression Purif..

[cit66] Andreassi J. L., Dabovic K., Leyh T. S. (2004). Biochemistry.

[cit67] Wang J., Niyompanich S., Tai Y.-S., Wang J., Bai W., Mahida P., Gao T., Zhang K. (2016). Appl. Environ. Microbiol..

[cit68] Redding-Johanson A. M., Batth T. S., Chan R., Krupa R., Szmidt H. L., Adams P. D., Keasling J. D., Lee T. S., Mukhopadhyay A., Petzold C. J. (2011). Metab. Eng..

[cit69] You S., Yin Q., Zhang J., Zhang C., Qi W., Gao L., Tao Z., Su R., He Z. (2017). Bioresour. Technol..

[cit70] Primak Y. A., Du M., Miller M. C., Wells D. H., Nielsen A. T., Weyler W., Beck Z. Q. (2011). Appl. Environ. Microbiol..

[cit71] Kazieva E., Yamamoto Y., Tajima Y., Yokoyama K., Katashkina J., Nishio Y. (2017). Microbiology.

[cit72] George K. W., Thompson M. G., Kang A., Baidoo E., Wang G., Chan L. J. G., Adams P. D., Petzold C. J., Keasling J. D., Lee T. S. (2015). Sci. Rep..

[cit73] Kim S.-J., Kim S. K., Seong W., Woo S.-G., Lee H., Yeom S.-J., Kim H., Lee D.-H., Lee S.-G. (2019). Catalysts.

[cit74] Li M., Chen H., Liu C., Guo J., Xu X., Zhang H., Nian R., Xian M. (2019). Microb. Cell Fact..

[cit75] Wu J., Cheng S., Cao J., Qiao J., Zhao G.-R. (2019). J. Agric. Food Chem..

[cit76] Whited G. M., Feher F. J., Benko D. A., Cervin M. A., Chotani G. K., McAuliffe J. C., LaDuca R. J., Ben-Shoshan E. A., Sanford K. J. (2010). Ind. Biotechnol..

[cit77] Chen H., Liu C., Li M., Zhang H., Xian M., Liu H. (2018). RSC Adv..

[cit78] Chen H., Li M., Liu C., Zhang H., Xian M., Liu H. (2018). Microb. Cell Fact..

[cit79] Dutoit R., De Ruyck J., Durisotti V., Legrain C., Jacobs E., Wouters J. (2008). Proteins.

[cit80] Siddiqui M. A., Yamanaka A., Hirooka K., Bamaba T., Kobayashi A., Imanaka T., Fukusaki E. I., Fujiwara S. (2005). Biochem. Biophys. Res. Commun..

[cit81] Yamashita S., Hemmi H., Ikeda Y., Nakayama T., Nishino T. (2004). Eur. J. Biochem..

[cit82] Kaneda K., Kuzuyama T., Takagi M., Hayakawa Y., Seto H. (2001). Proc. Natl. Acad. Sci. U. S. A..

[cit83] Laupitz R., Hecht S., Amslinger S., Zepeck F., Kaiser J., Richter G., Schramek N., Steinbacher S., Huber R., Arigoni D. (2004). Eur. J. Biochem..

[cit84] Barkley S. J., Cornish R. M., Poulter C. D. (2004). J. Bacteriol..

[cit85] Yang J., Xian M., Su S., Zhao G., Nie Q., Jiang X., Zheng Y., Liu W. (2012). PLoS One.

[cit86] Yoon S. H., Lee S. H., Das A., Ryu H. K., Jang H. J., Kim J. Y., Oh D. K., Keasling J. D., Kim S. W. (2009). J. Biotechnol..

[cit87] Zurbriggen A., Kirst H., Melis A. (2012). BioEnergy Res..

[cit88] Nybo S. E., Saunders J., McCormick S. P. (2017). J. Biotechnol..

[cit89] Morrone D., Lowry L., Determan M. K., Hershey D. M., Xu M., Peters R. J. (2010). Appl. Microbiol. Biotechnol..

[cit90] Jang H.-J., Yoon S.-H., Ryu H.-K., Kim J.-H., Wang C.-L., Kim J.-Y., Oh D.-K., Kim S.-W. (2011). Microb. Cell Fact..

[cit91] Li Z., Chen Q., Tang J., Li Q., Zhang X. (2019). Chin. J. Biotechnol..

[cit92] Yang C., Gao X., Jiang Y., Sun B., Gao F., Yang S. (2016). Metab. Eng..

[cit93] Kirby J., Dietzel K. L., Wichmann G., Chan R., Antipov E., Moss N., Baidoo E. E. K., Jackson P., Gaucher S. P., Gottlieb S., LaBarge J., Mahatdejkul T., Hawkins K. M., Muley S., Newman J. D., Liu P., Keasling J. D., Zhao L. (2016). Metab. Eng..

[cit94] Rising K. A., Crenshaw C. M., Koo H. J., Subramanian T., Chehade K. A. H., Starks C., Allen K. D., Andres D. A., Spielmann H. P., Noel J. P., Chappell J. (2015). ACS Chem. Biol..

[cit95] Kirby J., Nishimoto M., Chow R. W., Baidoo E. E., Wang G., Martin J., Schackwitz W., Chan R., Fortman J. L., Keasling J. D. (2015). Appl. Environ. Microbiol..

[cit96] Perez-Gil J., Uros E. M., Sauret-Güeto S., Lois L. M., Kirby J., Nishimoto M., Baidoo E. E., Keasling J. D., Boronat A., Rodriguez-Concepcion M. (2012). PLoS One.

[cit97] King J. R., Woolston B. M., Stephanopoulos G. (2017). ACS Synth. Biol..

[cit98] Erb T. J., Evans B. S., Cho K., Warlick B. P., Sriram J., Wood B. M., Imker H. J., Sweedler J. V., Tabita F. R., Gerlt J. A. (2012). Nat. Chem. Biol..

[cit99] Warlick B. P., Evans B. S., Erb T. J., Ramagopal U. A., Sriram J., Imker H. J., Sauder J. M., Bonanno J. B., Burley S. K., Tabita F. R. (2012). Biochemistry.

[cit100] Miller A. R., North J. A., Wildenthal J. A., Tabita F. R. (2018). mBio.

[cit101] North J. A., Wildenthal J. A., Erb T. J., Evans B. S., Byerly K. M., Gerlt J. A., Tabita F. R. (2020). Mol. Microbiol..

[cit102] Domenech C. E., Giordano W., Ávalos J., Cerdá-olmedo E. (1996). Eur. J. Biochem..

[cit103] Mahmud T., Wenzel S. C., Wan E., Wen K. W., Bode H. B., Gaitatzis N., Müller R. (2005). ChemBioChem.

[cit104] Ginger M. L., Chance M. L., Sadler I. H., Goad L. J. (2001). J. Biol. Chem..

[cit105] Yamauchi N. (2010). Biosci., Biotechnol., Biochem..

[cit106] Ginger M. L., Chance M. L., Goad L. J. (1999). Biochem. J..

[cit107] Grochowski L. L., Xu H., White R. H. (2006). J. Bacteriol..

[cit108] Dellas N., Thomas S. T., Manning G., Noel J. P. (2013). eLife.

[cit109] VanNice J. C., Skaff D. A., Keightley A., Addo J. K., Wyckoff G. J., Miziorko H. M. (2014). J. Bacteriol..

[cit110] Beck Z. Q. , Mampel J., Meurer G., Miller M. C., Sanford K. J., Vaviline D. V., Weyler W. and Whited G. M., United States Pat., US20160002672A1, 2016

[cit111] Azami Y., Hattori A., Nishimura H., Kawaide H., Yoshimura T., Hemmi H. (2014). J. Biol. Chem..

[cit112] Vinokur J. M., Korman T. P., Cao Z., Bowie J. U. (2014). Biochemistry.

[cit113] Vinokur J. M., Cummins M. C., Korman T. P., Bowie J. U. (2016). Sci. Rep..

[cit114] Vinokur J. M., Korman T. P., Sawaya M. R., Collazo M., Cascio D., Bowie J. U. (2015). Protein Sci..

[cit115] De Waard A., Phillips A., Bloch K. (1959). J. Am. Chem. Soc..

[cit116] Lindberg M., Yuan C., Dewaard A., Bloch K. (1962). Biochemistry.

[cit117] Hayakawa H., Sobue F., Motoyama K., Yoshimura T., Hemmi H. (2017). Biochem. Biophys. Res. Commun..

[cit118] Marliere P. , Anissimova M., Chayot R. and Delcourt M., WIPO Pat., WO2011076261, 2011

[cit119] Motoyama K., Sobue F., Kawaide H., Yoshimura T., Hemmi H. (2019). Appl. Environ. Microbiol..

[cit120] Hayakawa H., Motoyama K., Sobue F., Ito T., Kawaide H., Yoshimura T., Hemmi H. (2018). Proc. Natl. Acad. Sci. U. S. A..

[cit121] Yoshida R., Yoshimura T., Hemmi H. (2020). Appl. Environ. Microbiol..

[cit122] Henry L. K., Thomas S. T., Widhalm J. R., Lynch J. H., Davis T. C., Kessler S. A., Bohlmann J., Noel J. P., Dudareva N. (2018). Nat. Plants.

[cit123] George K. W., Thompson M. G., Kim J., Baidoo E. E. K., Wang G., Benites V. T., Petzold C. J., Chan L. J. G., Yilmaz S., Turhanen P., Adams P. D., Keasling J. D., Lee T. S. (2018). Metab. Eng..

[cit124] Yang J., Nie Q., Liu H., Xian M., Liu H. (2016). BMC Biotechnol..

[cit125] Kang A., George K. W., Wang G., Baidoo E., Keasling J. D., Lee T. S. (2016). Metab. Eng..

[cit126] Kang A., Meadows C. W., Canu N., Keasling J. D., Lee T. S. (2017). Metab. Eng..

[cit127] Kang A., Mendez-Perez D., Goh E.-B., Baidoo E. E., Benites V. T., Beller H. R., Keasling J. D., Adams P. D., Mukhopadhyay A., Lee T. S. (2019). Metab. Eng..

[cit128] Gao Y., Honzatko R. B., Peters R. J. (2012). Nat. Prod. Rep..

[cit129] Chatzivasileiou A. O., Ward V., Edgar S. M., Stephanopoulos G. (2019). Proc. Natl. Acad. Sci. U. S. A..

[cit130] Clomburg J. M., Qian S., Tan Z., Cheong S., Gonzalez R. (2019). Proc. Natl. Acad. Sci. U. S. A..

[cit131] Couillaud J., Rico J., Rubini A., Hamrouni T., Courvoisier-Dezord E., Petit J.-L., Mariage A., Darii E., Duquesne K., de Berardinis V. (2019). ACS Omega.

[cit132] Liu Y., Yan Z., Lu X., Xiao D., Jiang H. (2016). Sci. Rep..

[cit133] Lund S., Hall R., Williams G. J. (2019). ACS Synth. Biol..

[cit134] Johnson L. A., Dunbabin A., Benton J. C. R., Mart R. J., Allemann R. K. (2020). Angew. Chem., Int. Ed..

[cit135] Li M., Hou F., Wu T., Jiang X., Li F., Liu H., Xian M., Zhang H. (2020). Nat. Prod. Rep..

[cit136] Rico J., Duquesne K., Petit J. L., Mariage A., Darii E., Peruch F., de Berardinis V., Iacazio G. (2019). Microb. Cell Fact..

[cit137] Ferraz C. A., Leferink N. G. H., Kosov I., Scrutton N. S. (2021). ChemBioChem.

[cit138] Alonso-Gutierrez J., Chan R., Batth T. S., Adams P. D., Keasling J. D., Petzold C. J., Lee T. S. (2013). Metab. Eng..

[cit139] Ruzicka L. (1953). Experientia.

[cit140] Schönherr H., Cernak T. (2013). Angew. Chem., Int. Ed..

[cit141] Dickschat J. S., Nawrath T., Thiel V., Kunze B., Müller R., Schulz S. (2007). Angew. Chem., Int. Ed..

[cit142] Komatsu M., Tsuda M., Omura S., Oikawa H., Ikeda H. (2008). Proc. Natl. Acad. Sci. U. S. A..

[cit143] Wang C. M., Cane D. E. (2008). J. Am. Chem. Soc..

[cit144] Ozaki T., Shinde S. S., Gao L., Okuizumi R., Liu C., Ogasawara Y., Lei X., Dairi T., Minami A., Oikawa H. (2018). Angew. Chem., Int. Ed..

[cit145] von Reuss S., Domik D., Lemfack M. C., Magnus N., Kai M., Weise T., Piechulla B. (2018). J. Am. Chem. Soc..

[cit146] Kschowak M. J., Wortmann H., Dickschat J. S., Schrader J., Buchhaupt M. (2018). PLoS One.

[cit147] Kschowak M. J., Maier F., Wortmann H., Buchhaupt M. (2020). ACS Synth. Biol..

[cit148] Drummond L., Kschowak M. J., Breitenbach J., Wolff H., Shi Y. M., Schrader J., Bode H. B., Sandmann G., Buchhaupt M. (2019). ACS Synth. Biol..

[cit149] Kobayashi M., Koyama T., Ogura K., Seto S., Ritter F. J., Briiggemann-Rotgans I. E. M. (1980). J. Am. Chem. Soc..

[cit150] Koyama T., Ogura K. (1987). J. Am. Chem. Soc..

[cit151] Attygalle A. B., Morgan E. D. (1982). J. Chem. Soc..

[cit152] Schooley D. A., Judy K. J., Bergot B. J., Hall M. S., Siddal J. B. (1973). Proc. Natl. Acad. Sci. U. S. A..

[cit153] Eiben C. B., de Rond T., Bloszies C., Gin J., Chiniquy J., Baidoo E. E. K., Petzold C. J., Hillson N. J., Fiehn O., Keasling J. D. (2019). ACS Synth. Biol..

[cit154] Reiling K. K., Yoshikuni Y., Martin V. J., Newman J., Bohlmann J., Keasling J. D. (2004). Biotechnol. Bioeng..

[cit155] Fujisaki S., Hara H., Nishimura Y., Horiuchi K., Nishino T. (1990). J. Biochem..

[cit156] Saiki K., Mogi T., Anraku Y. (1992). Biochem. Biophys. Res. Commun..

[cit157] Okada K., Minehira M., Zhu X., Suzuki K., Nakagawa T., Matsuda H., Kawamukai M. (1997). J. Bacteriol..

[cit158] Apfel C. M., Takács B., Fountoulakis M., Stieger M., Keck W. (1999). J. Bacteriol..

[cit159] Wang X., Wu J., Chen J., Xiao L., Li X. (2019). J. Agric. Food Chem..

[cit160] Rolf J., Julsing M. K., Rosenthal K., Lütz S. (2020). Molecules.

[cit161] Zhang H., Liu Q., Cao Y., Feng X., Zheng Y., Zou H., Liu H., Yang J., Xian M. (2014). Microb. Cell Fact..

[cit162] Liu W., Xu X., Zhang R., Cheng T., Cao Y., Li X., Guo J., Liu H., Xian M. (2016). Biotechnol. Biofuels.

[cit163] Chacón M. G., Marriott A., Kendrick E. G., Styles M. Q., Leak D. J. (2019). Microb. Cell Fact..

[cit164] Yang J., Nie Q., Ren M., Feng H., Jiang X., Zheng Y., Liu M., Zhang H., Xian M. (2013). Biotechnol. Biofuels.

[cit165] Mendez-Perez D., Alonso-Gutierrez J., Hu Q., Molinas M., Baidoo E. E., Wang G., Chan L. J., Adams P. D., Petzold C. J., Keasling J. D. (2017). Biotechnol. Bioeng..

[cit166] Kim E.-M., Eom J.-H., Um Y., Kim Y., Woo H. M. (2015). J. Agric. Food Chem..

[cit167] Burke C., Croteau R. (2002). Arch. Biochem. Biophys..

[cit168] Tashiro M., Kiyota H., Kawai-Noma S., Saito K., Ikeuchi M., Iijima Y., Umeno D. (2016). ACS Synth. Biol..

[cit169] Sarria S., Wong B., Martín H. G., Keasling J. D., Peralta-Yahya P. (2014). ACS Synth. Biol..

[cit170] Wang X., Chen J., Zhang J., Zhou Y., Zhang Y., Wang F., Li X. (2021). Metab. Eng..

[cit171] Willrodt C., David C., Cornelissen S., Bühler B., Julsing M. K., Schmid A. (2014). Biotechnol. J..

[cit172] Shukal S., Chen X., Zhang C. (2019). Metab. Eng..

[cit173] Dietrich J. A., Yoshikuni Y., Fisher K. J., Woolard F. X., Ockey D., McPhee D. J., Renninger N. S., Chang M. C., Baker D., Keasling J. D. (2009). ACS Chem. Biol..

[cit174] Chang M. C., Eachus R. A., Trieu W., Ro D.-K., Keasling J. D. (2007). Nat. Chem. Biol..

[cit175] Yao P., You S., Qi W., Su R., He Z. (2020). Environ. Sci. Pollut. Res..

[cit176] Zhu F., Zhong X., Hu M., Lu L., Deng Z., Liu T. (2014). Biotechnol. Bioeng..

[cit177] Wang C., Park J.-E., Choi E.-S., Kim S.-W. (2016). Biotechnol. J..

[cit178] Zhang C., Seow V. Y., Chen X., Too H.-P. (2018). Nat. Commun..

[cit179] Aguilar F., Scheper T., Beutel S. (2019). Molecules.

[cit180] Luo L.-Q., Chen Y.-G., Li D.-S., Liu Y., Li S.-H. (2020). Chem. Biodiversity.

[cit181] Xie X., Kirby J., Keasling J. D. (2012). Phytochemistry.

[cit182] Cao Y., Zhang R., Liu W., Zhao G., Niu W., Guo J., Xian M., Liu H. (2019). Sci. Rep..

[cit183] Cheng T., Zhao G., Xian M., Xie C. (2020). Sci. Rep..

[cit184] Mou S.-B., Xiao W., Wang H.-Q., Chen K.-Y., Xiang Z. (2020). Org. Lett..

[cit185] Schalk M., Pastore L., Mirata M. A., Khim S., Schouwey M., Deguerry F., Pineda V., Rocci L., Daviet L. (2012). J. Am. Chem. Soc..

[cit186] Leonard E., Ajikumar P. K., Thayer K., Xiao W.-H., Mo J. D., Tidor B., Stephanopoulos G., Prather K. L. (2010). Proc. Natl. Acad. Sci. U. S. A..

[cit187] Wang J.-F., Xiong Z.-Q., Li S.-Y., Wang Y. (2013). Appl. Microbiol. Biotechnol..

[cit188] Matsuba Y., Zi J., Jones A. D., Peters R. J., Pichersky E. (2015). PLoS One.

[cit189] van der Hoeven R. S., Monforte A. J., Breeden D., Tanksley S. D., Steffens J. C. (2000). Plant Cell.

[cit190] Sallaud C., Rontein D., Onillon S., Jabès F., Duffé P., Giacalone C., Thoraval S., Escoffier C., Herbette G., Leonhardt N. (2009). Plant Cell.

[cit191] Zi J., Matsuba Y., Hong Y. J., Jackson A. J., Tantillo D. J., Pichersky E., Peters R. J. (2014). J. Am. Chem. Soc..

[cit192] Akhtar T. A., Matsuba Y., Schauvinhold I., Yu G., Lees H. A., Klein S. E., Pichersky E. (2013). Plant J..

[cit193] Gonzales-Vigil E., Hufnagel D. E., Kim J., Last R. L., Barry C. S. (2012). Plant J..

[cit194] Falara V., Akhtar T. A., Nguyen T. T., Spyropoulou E. A., Bleeker P. M., Schauvinhold I., Matsuba Y., Bonini M. E., Schilmiller A. L., Last R. L. (2011). Plant Physiol..

[cit195] Schilmiller A. L., Schauvinhold I., Larson M., Xu R., Charbonneau A. L., Schmidt A., Wilkerson C., Last R. L., Pichersky E. (2009). Proc. Natl. Acad. Sci. U. S. A..

[cit196] Zhang M., Liu J., Li K., Yu D. (2013). PLoS One.

[cit197] Gericke O., Hansen N. L., Pedersen G. B., Kjaerulff L., Luo D., Staerk D., Møller B. L., Pateraki I., Heskes A. M. (2020). BMC Plant Biol..

[cit198] Ignea C., Raadam M. H., Motawia M. S., Makris A. M., Vickers C. E., Kampranis S. C. (2019). Nat. Commun..

[cit199] Cheng S., Liu X., Jiang G., Wu J., Zhang J.-l., Lei D., Yuan Y.-J., Qiao J., Zhao G.-R. (2019). ACS Synth. Biol..

[cit200] Johnson S. R., Bhat W. W., Sadre R., Miller G. P., Garcia A. S., Hamberger B. (2019). New Phytol..

[cit201] Jia Q., Li G., Köllner T. G., Fu J., Chen X., Xiong W., Crandall-Stotler B. J., Bowman J. L., Weston D. J., Zhang Y. (2016). Proc. Natl. Acad. Sci. U. S. A..

[cit202] Jia M., Peters R. J. (2017). Org. Biomol. Chem..

[cit203] Pelot K. A., Hagelthorn D. M., Hong Y. J., Tantillo D. J., Zerbe P. (2019). ChemBioChem.

[cit204] Cao X., Lv Y.-B., Chen J., Imanaka T., Wei L.-J., Hua Q. (2016). Biotechnol. Biofuels.

[cit205] Wang C., Zhou J., Jang H.-J., Yoon S.-H., Kim J.-Y., Lee S.-G., Choi E.-S., Kim S.-W. (2013). Metab. Eng..

[cit206] Oldfield E., Lin F. Y. (2012). Angew. Chem., Int. Ed..

[cit207] Lee J. S., Pan J. J., Ramamoorthy G., Poulter C. D. (2017). J. Am. Chem. Soc..

[cit208] Zhang Y., Nielsen J., Liu Z. (2017). FEMS Yeast Res..

[cit209] Demissie Z. A., Erland L. A., Rheault M. R., Mahmoud S. S. (2013). J. Biol. Chem..

[cit210] Teufel R. (2018). Methods Enzymol..

[cit211] Liu M., Chen C. C., Chen L., Xiao X., Zheng Y., Huang J. W., Liu W., Ko T. P., Cheng Y. S., Feng X., Oldfield E., Guo R. T., Ma Y. (2016). Angew. Chem., Int. Ed..

[cit212] Rivera S. B., Swedlund B. D., King G. J., Bell R. N., Hussey C. E., Shattuck-Eidens D. M., Wrobel W. M., Peiser G. D., Poulter C. D. (2001). Proc. Natl. Acad. Sci. U. S. A..

[cit213] Thulasiram H. V., Erickson H. K., Poulter C. D. (2008). J. Am. Chem. Soc..

[cit214] He H., Bian G., Herbst-Gervasoni C. J., Mori T., Shinsky S. A., Hou A., Mu X., Huang M., Cheng S., Deng Z., Christianson D. W., Abe I., Liu T. (2020). Nat. Commun..

[cit215] Chekan J. R., McKinnie S. M. K., Noel J. P., Moore B. S. (2020). Proc. Natl. Acad. Sci. U. S. A..

[cit216] Koyama T., Saito A., Ogura K., Seto S. (1980). J. Am. Chem. Soc..

[cit217] Nagaki M., Yamamoto H., Takahashi A., Maki Y., Ishibashi J., Nishino T., Koyama T. (2002). J. Mol. Catal. B: Enzym..

[cit218] Maki Y., Komabayashi M., Gotoh Y., Ohya N., Hemmi H., Hirooka K., Nishino T., Koyama T. (2002). J. Mol. Catal. B: Enzym..

[cit219] Heaps N. A., Poulter C. D. (2011). J. Org. Chem..

[cit220] Harms V., Kirschning A., Dickschat J. S. (2020). Nat. Prod. Rep..

[cit221] Braun P., LaBaer J. (2003). Trends Biotechnol..

[cit222] Yesilirmak F., Sayers Z. (2009). Int. J. Plant Genomics.

[cit223] Soliman S., Tang Y. (2015). Biotechnol. Bioeng..

[cit224] Leferink N. G., Jervis A. J., Zebec Z., Toogood H. S., Hay S., Takano E., Scrutton N. S. (2016). ChemistrySelect.

[cit225] Karuppiah V., Ranaghan K. E., Leferink N. G., Johannissen L. O., Shanmugam M., Ní Cheallaigh A., Bennett N. J., Kearsey L. J., Takano E., Gardiner J. M. (2017). ACS Catal..

[cit226] Karuppiah V. , Leferink N. G. and Scrutton N. S., WIPO Pat., WO2018142109A1, 2020

[cit227] Zhang C., Chen X., Lee R. T. C., T R., Maurer-Stroh S., Rühl M. (2021). Commun. Biol..

[cit228] Thanasomboon R., Waraho D., Cheevadhanarak S., Meechai A. (2012). Procedia Computer Science.

[cit229] Wu W., Liu F., Davis R. W. (2018). Macromol. Rapid Commun..

[cit230] Zhou P., Du Y., Xu N., Yue C., Ye L. (2020). Biochem. Eng. J..

[cit231] Liu C.-L., Tian T., Alonso-Gutierrez J., Garabedian B., Wang S., Baidoo E. E., Benites V., Chen Y., Petzold C. J., Adams P. D. (2018). Biotechnol. Biofuels.

[cit232] Leonard E., Koffas M. A. G. (2007). Appl. Environ. Microbiol..

[cit233] Zelasko S., Palaria A., Das A. (2013). Protein Expression Purif..

[cit234] Janocha S. , Schmitz D. and Bernhardt R., in Biotechnology of Isoprenoids, Springer, 2015, pp. 215–250

[cit235] Biggs B. W., Lim C. G., Sagliani K., Shankar S., Stephanopoulos G., De Mey M., Ajikumar P. K. (2016). Proc. Natl. Acad. Sci. U. S. A..

[cit236] Harada H., Shindo K., Iki K., Teraoka A., Okamoto S., Yu F., Hattan J. I., Utsumi R., Misawa N. (2011). Appl. Microbiol. Biotechnol..

[cit237] Schifrin A., Khatri Y., Kirsch P., Thiel V., Schulz S., Bernhardt R. (2016). Org. Biomol. Chem..

[cit238] Rabe P., Dickschat J. S. (2013). Angew. Chem., Int. Ed..

[cit239] Agger S. A., Lopez-Gallego F., Hoye T. R., Schmidt-Dannert C. (2008). J. Bacteriol..

[cit240] Reddy G. K., Leferink N. G. H., Umemura M., Ahmed S. T., Breitling R., Scrutton N. S., Takano E. (2020). PLoS One.

[cit241] Rabe P., Schmitz T., Dickschat J. S. (2016). Beilstein J. Org. Chem..

[cit242] Yamada Y., Kuzuyama T., Komatsu M., Shin-ya K., Omura S., Cane D. E., Ikeda H. (2015). Proc. Natl. Acad. Sci. U. S. A..

[cit243] Giglio S., Chou W., Ikeda H., Cane D., Monis P. (2011). Environ. Sci. Technol..

[cit244] Komatsu M., Tsuda M., Ōmura S., Oikawa H., Ikeda H. (2008). Proc. Natl. Acad. Sci. U. S. A..

[cit245] Dickschat J. S. (2016). Nat. Prod. Rep..

[cit246] Dickschat J. S. (2019). Angew. Chem., Int. Ed..

[cit247] Helfrich E. J. N., Lin G.-M., Voigt C. A., Clardy J. (2019). Beilstein J. Org. Chem..

[cit248] Tomm H. A., Ucciferri L., Ross A. C. (2019). J. Ind. Microbiol. Biotechnol..

[cit249] Cimermancic P., Medema M. H., Claesen J., Kurita K., Brown L. C. W., Mavrommatis K., Pati A., Godfrey P. A., Koehrsen M., Clardy J. (2014). Cell.

[cit250] Ziemert N., Weber T., Medema M. H. (2020). Reference Module in Chemistry, Molecular Sciences and Chemical Engineering.

[cit251] Blin K., Pascal Andreu V., de los Santos E. L. C., Del Carratore F., Lee S. Y., Medema M. H., Weber T. (2019). Nucleic Acids Res..

[cit252] Medema M. H., Kottmann R., Yilmaz P., Cummings M., Biggins J. B., Blin K., De Bruijn I., Chooi Y. H., Claesen J., Coates R. C. (2015). Nat. Chem. Biol..

[cit253] Huo L., Hug J. J., Fu C., Bian X., Zhang Y., Müller R. (2019). Nat. Prod. Rep..

[cit254] Zhang X., Hindra, Elliot M. A. (2019). Curr. Opin. Microbiol..

[cit255] Palazzotto E., Weber T. (2018). Curr. Opin. Microbiol..

[cit256] Robinson C. J., Carbonell P., Jervis A. J., Yan C., Hollywood K. A., Dunstan M. S., Currin A., Swainston N., Spiess R., Taylor S., Mulherin P., Parker S., Rowe W., Matthews N. E., Malone K. J., Le Feuvre R., Shapira P., Barran P., Turner N. J., Micklefield J., Breitling R., Takano E., Scrutton N. S. (2020). Metab. Eng..

[cit257] Leferink N. G., Dunstan M. S., Hollywood K. A., Swainston N., Currin A., Jervis A. J., Takano E., Scrutton N. S. (2019). Sci. Rep..

[cit258] Sikkema J., de Bont J. A., Poolman B. (1995). Microbiol. Mol. Biol. Rev..

[cit259] Sikkema J., de Bont J. A., Poolman B. (1994). J. Biol. Chem..

[cit260] Heipieper H. and Martínez P., in Handbook of hydrocarbon and lipid microbiology, 2010

[cit261] Doshi R., Nguyen T., Chang G. (2013). Proc. Natl. Acad. Sci. U. S. A..

[cit262] Griffin S. G., Wyllie S. G., Markham J. L., Leach D. N. (1999). Flavour Fragrance J..

[cit263] Vermaas J. V., Bentley G. J., Beckham G. T., Crowley M. F. (2018). J. Phys. Chem. B.

[cit264] Chubukov V., Mingardon F., Schackwitz W., Baidoo E. E., Alonso-Gutierrez J., Hu Q., Lee T. S., Keasling J. D., Mukhopadhyay A. (2015). Appl. Environ. Microbiol..

[cit265] Shah A. A., Wang C., Yoon S.-H., Kim J.-Y., Choi E.-S., Kim S.-W. (2013). J. Biotechnol..

[cit266] Paddon C. J., Westfall P. J., Pitera D. J., Benjamin K., Fisher K., McPhee D., Leavell M., Tai A., Main A., Eng D. (2013). Nature.

[cit267] Newman J. D., Marshall J., Chang M., Nowroozi F., Paradise E., Pitera D., Newman K. L., Keasling J. D. (2006). Biotechnol. Bioeng..

[cit268] Jones C. M., Lozada N. J. H., Pfleger B. F. (2015). Appl. Microbiol. Biotechnol..

[cit269] Piddock L. J. V. (2006). Clin. Microbiol. Rev..

[cit270] Zhang C., Chen X., Stephanopoulos G., Too H. P. (2016). Biotechnol. Bioeng..

[cit271] Niu F.-X., He X., Wu Y.-Q., Liu J.-Z. (2018). Frontiers in Microbiology.

[cit272] Weston N., Sharma P., Ricci V., Piddock L. J. V. (2018). Res. Microbiol..

[cit273] Shah A. A., Wang C., Chung Y.-R., Kim J.-Y., Choi E.-S., Kim S.-W. (2013). J. Biosci. Bioeng..

[cit274] Foo J. L., Jensen H. M., Dahl R. H., George K., Keasling J. D., Lee T. S., Leong S., Mukhopadhyay A. (2014). mBio.

[cit275] Dunlop M. J., Dossani Z. Y., Szmidt H. L., Chu H. C., Lee T. S., Keasling J. D., Hadi M. Z., Mukhopadhyay A. (2011). Mol. Syst. Biol..

[cit276] Turner W. J., Dunlop M. J. (2015). ACS Synth. Biol..

[cit277] Meng Y., Shao X., Wang Y., Li Y., Zheng X., Wei G., Kim S. W., Wang C. (2020). Biotechnol. Bioeng..

[cit278] Wu T., Ye L., Zhao D., Li S., Li Q., Zhang B., Bi C., Zhang X. (2017). Metab. Eng..

[cit279] Wu T., Li S., Ye L., Zhao D., Fan F., Li Q., Zhang B., Bi C., Zhang X. (2019). ACS Synth. Biol..

[cit280] Wu T., Liu J., Li M., Zhang G., Liu L., Li X., Men X., Xian M., Zhang H. (2020). Biotechnol. Biofuels.

[cit281] Tomko T. A., Dunlop M. J. (2015). Biotechnol. Biofuels.

[cit282] Sattayawat P., Yunus I. S., Jones P. R. (2020). Proc. Natl. Acad. Sci. U. S. A..

[cit283] Carbonell P., Parutto P., Baudier C., Junot C., Faulon J.-L. (2014). ACS Synth. Biol..

[cit284] Carbonell P., Wong J., Swainston N., Takano E., Turner N. J., Scrutton N. S., Kell D. B., Breitling R., Faulon J.-L. (2018). Bioinformatics.

[cit285] Edgar S., Li F.-S., Qiao K., Weng J.-K., Stephanopoulos G. (2017). ACS Synth. Biol..

[cit286] Lv X., Gu J., Wang F., Xie W., Liu M., Ye L., Yu H. (2016). Biotechnol. Bioeng..

[cit287] Swainston N., Dunstan M., Jervis A. J., Robinson C. J., Carbonell P., Williams A. R., Faulon J.-L., Scrutton N. S., Kell D. B. (2018). Bioinformatics.

[cit288] Jervis A. J., Carbonell P., Taylor S., Sung R., Dunstan M. S., Robinson C. J., Breitling R., Takano E., Scrutton N. S. (2019). ACS Synth. Biol..

[cit289] Jervis A. J., Carbonell P., Vinaixa M., Dunstan M. S., Hollywood K. A., Robinson C. J., Rattray N. J., Yan C., Swainston N., Currin A. (2018). ACS Synth. Biol..

[cit290] Carbonell P., Jervis A. J., Robinson C. J., Yan C., Dunstan M., Swainston N., Vinaixa M., Hollywood K. A., Currin A., Rattray N. J. (2018). Commun. Biol..

[cit291] Otero-Muras I., Carbonell P. (2020). Metab. Eng..

[cit292] Lawson C. E., Martí J. M., Radivojevic T., Jonnalagadda S. V. R., Gentz R., Hillson N. J., Peisert S., Kim J., Simmons B. A., Petzold C. J., Singer S. W., Mukhopadhyay A., Tanjore D., Dunn J. G., Garcia Martin H. (2020). Metab. Eng..

[cit293] Emmerstorfer-Augustin A., Moser S., Pichler H. (2016). J. Biotechnol..

[cit294] Farmer W. R., Liao J. C. (2001). Biotechnol. Prog..

[cit295] Jung J., Lim J. H., Kim S. Y., Im D.-K., Seok J. Y., Lee S.-J. V., Oh M.-K., Jung G. Y. (2016). Metab. Eng..

[cit296] Liu H., Sun Y., Ramos K. R. M., Nisola G. M., Valdehuesa K. N. G., Lee W. K., Park S. J., Chung W.-J. (2013). PLoS One.

[cit297] Ng C. Y., Farasat I., Maranas C. D., Salis H. M. (2015). Metab. Eng..

[cit298] Ramos K. R. M., Valdehuesa K. N. G., Liu H., Nisola G. M., Lee W.-K., Chung W.-J. (2014). Bioprocess Biosyst. Eng..

[cit299] Zhao J., Li Q., Sun T., Zhu X., Xu H., Tang J., Zhang X., Ma Y. (2013). Metab. Eng..

[cit300] Sun T., Miao L., Li Q., Dai G., Lu F., Liu T., Zhang X., Ma Y. (2014). Biotechnol. Lett..

[cit301] Becker J., Wittmann C. (2016). Curr. Opin. Biotechnol..

[cit302] Bokinsky G., Peralta-Yahya P. P., George A., Holmes B. M., Steen E. J., Dietrich J., Soon Lee T., Tullman-Ercek D., Voigt C. A., Simmons B. A., Keasling J. D. (2011). Proc. Natl. Acad. Sci. U. S. A..

[cit303] Scullin C., Stavila V., Skarstad A., Keasling J. D., Simmons B. A., Singh S. (2015). Bioresour. Technol..

[cit304] Shi J., George K. W., Sun N., He W., Li C., Stavila V., Keasling J. D., Simmons B. A., Lee T. S., Singh S. (2015). BioEnergy Res..

[cit305] Davies F. K., Jinkerson R. E., Posewitz M. C. (2015). Photosynth. Res..

[cit306] Gao X., Gao F., Liu D., Zhang H., Nie X., Yang C. (2016). Energy Environ. Sci..

[cit307] Wu J., Wang X., Xiao L., Wang F., Zhang Y., Li X. (2021). J. Agric. Food Chem..

[cit308] Wang X., Pereira J. H., Tsutakawa S., Fang X., Adams P. D., Mukhopadhyay A., Lee T. S. (2021). Metab. Eng..

[cit309] Han G. H., Kim S. K., Yoon P. K.-S., Kang Y., Kim B. S., Fu Y., Sung B. H., Jung H. C., Lee D.-H., Kim S.-W., Lee S.-G. (2016). Microb. Cell Fact..

[cit310] Zada B., Wang C., Park J.-B., Jeong S.-H., Park J.-E., Singh H. B., Kim S.-W. (2018). Biotechnol. Biofuels.

[cit311] Yang L., Wang C., Zhou J., Kim S.-W. (2016). Microb. Cell Fact..

[cit312] Siemon T., Wang Z., Bian G., Seitz T., Ye Z., Lu Y., Cheng S., Ding Y., Huang Y., Deng Z., Liu T., Christmann M. (2020). J. Am. Chem. Soc..

[cit313] Alemdar S., König J. C., Hartwig S., Frister T., Scheper T., Beutel S. (2017). Eng. Life Sci..

[cit314] Aguilar F., Ekramzadeh K., Scheper T., Beutel S. (2020). ACS Omega.

[cit315] Li D., Zhang Q., Zhou Z., Zhao F., Lu W. (2016). Biotechnol. Lett..

[cit316] Gong Z., Wang H., Tang J., Bi C., Li Q., Zhang X. (2020). J. Agric. Food Chem..

[cit317] Shen H.-J., Cheng B.-Y., Zhang Y.-M., Tang L., Li Z., Bu Y.-F., Li X.-R., Tian G.-Q., Liu J.-Z. (2016). Metab. Eng..

[cit318] Zhang C., Chen X., Lindley N. D., Too H. P. (2018). Biotechnol. Bioeng..

[cit319] Jang H.-J., Ha B.-K., Zhou J., Ahn J., Yoon S.-H., Kim S.-W. (2015). Biotechnol. Bioeng..

[cit320] Huang M., Wang Y., Liu J., Mao Z. (2011). Chin. J. Chem. Eng..

[cit321] Vuuren S. v., Viljoen A. M. (2007). Flavour Fragrance J..

[cit322] Peralta-Yahya P. P., Ouellet M., Chan R., Mukhopadhyay A., Keasling J. D., Lee T. S. (2011). Nat. Commun..

